# Does a provider payment method affect membership retention in a health insurance scheme? a mixed method study of Ghana’s capitation payment for primary care

**DOI:** 10.1186/s12913-018-2859-6

**Published:** 2018-01-30

**Authors:** Francis-Xavier Andoh-Adjei, Renske van der Wal, Eric Nsiah-Boateng, Felix Ankomah Asante, Koos van der Velden, Ernst Spaan

**Affiliations:** 1 NHIA, PMB Ministries Post Office, 36-6th Avenue, Ridge, Accra, Ghana; 2 Radboud Institute for Health Sciences, Department for Health Evidence, Radboud University Medical Centre-Netherlands, Nijmegen, Netherlands; 3 Institute of Statistical, Social and Economic Research (ISSER) University of Ghana, Legon-, Accra, Ghana; 4 Radboud Institute for Health Science, Department for Primary and Community Health, Radboud University Medical Centre-Netherlands, Nijmegen, Netherlands

**Keywords:** Capitation payment, Membership retention, Health insurance, Ghana

## Abstract

**Background:**

Ghana introduced a National Health Insurance Scheme (NHIS) in 2003 applying fee-for-service method for paying NHIS-credentialed health care providers. The National Health Insurance Authority (NHIA) later introduced diagnosis-related-grouping (DRG) payment to contain cost without much success. The NHIA then introduced capitation payment, a decision that attracted complaints of falling enrolment and renewal rates from stakeholders. This study was done to provide evidence on this trend to guide policy debate on the issue.

**Methods:**

We applied mixed method design to the study. We did a trend analysis of NHIS membership data in Ashanti, Volta and Central regions to assess growth rate; performed independent-sample *t*-test to compare sample means of the three regions and analysed data from individual in-depth interviews to determine any relationship between capitation payment and subscribers’ renewal decision.

**Results:**

Results of new enrolment data analysis showed differences in mean growth rates between Ashanti (*M* = 30.15, *SE* 3.03) and Volta (*M* = 40.72, *SE* 3.10), *p* = 0.041; *r* = 0. 15; and between Ashanti and Central (*M* = 47.38, *SE*6.49) *p* = 0.043; r = 0. 42. Analysis of membership renewal data, however, showed no significant differences in mean growth rates between Ashanti (*M* = 65.47, *SE* 6.67) and Volta (*M* = 69.29, *SE* 5.04), *p* = 0.660; *r* = 0.03; and between Ashanti and Central (*M* = 50.51, *SE* 9.49), *p* = 0.233. Analysis of both new enrolment and renewal data also showed no significant differences in mean growth rates between Ashanti (*M* = − 13.76, *SE* 17.68) and Volta (*M* = 5.48, *SE* 5.50), *p* = 0.329; and between Ashanti and Central (*M* = − 6.47, *SE* 12.68), *p* = 0.746. However, capitation payment had some effect in Ashanti compared with Volta (*r* = 0. 12) and Central (r = 0. 14); but could not be sustained beyond 2012. Responses from the in-depth interviews did not also show that capitation payment is a key factor in subscribers’ renewal decision.

**Conclusion:**

Capitation payment had a small but unsustainable effect on membership growth rate in the Ashanti region. Factors other than capitation payment may have played a more significant role in subscribers’ enrolment and renewal decisions in the Ashanti region of Ghana.

**Electronic supplementary material:**

The online version of this article (10.1186/s12913-018-2859-6) contains supplementary material, which is available to authorized users.

## Background

Countries all over the world face challenges with funding their health care systems [1] and as they strive to attain universal health coverage a two-pronged approach to addressing the funding challenge may be adopted: income side and expenditure side interventions. On the income-side, many low and middle-income countries are experimenting with new and innovative approaches, with health insurance becoming the new paradigm [2]. However, since no country in the world has unlimited funds for their health sector, policy-makers complement the income-side interventions with expenditure side interventions while being conscious of quality care delivery [3]. One such expenditure-side intervention is reform of the provider payment systems [4] which is intended to influence efficient application of resources in health care delivery [5]. Provider payment reform is well-documented as crucial for determining performance of health care systems and institutions in terms of cost containment, efficiency and quality of care [6, 7]. Since provider payment methods have their strengths and weaknesses, a growing number of developed and developing countries are grappling with a range of mixed payment methods by leveraging their strengths to ensure cost-efficiency and quality improvement in their health systems [6, 8, 9]. Over the last decade, countries have been reforming their provider payment systems to improve access to health services and control increasing cost of health care [7, 10]. Ghana introduced a National Health Insurance Scheme (NHIS) in 2003. Initially, the National Health Insurance Authority (NHIA) paid its credentialed providers by fee-for-service (FFS) method for both clinical services and medicine costs but had to switch to diagnosis-related-grouping (DRG) payment due to abuse of the system [11]. The DRG is used to pay for clinical services while the FFS payment is used to pay for medicines. Four years into the implementation of the DRG payment, studies show that high rate of health services utilization and escalating cost of claims still persist [11]. After careful consideration of these challenges facing the NHIS, the NHIA decided to pilot capitation payment as an alternative payment method for primary out-patients’ care in 2012 beginning with a pilot in the Ashanti region of Ghana. The objectives for the capitation payment were to contain cost by sharing financial risk among schemes, providers and subscribers; introduce managed competition among providers provide patients with the opportunity to choose their preferred primary care provider (PPP), improve efficiency and effectiveness of health service delivery through more rational resource use, correct the adverse effects of the G-DRG and to address difficulties in forecasting and budgeting.

### Membership registration and renewal

In theory, the law makes it mandatory for residents of Ghana to belong to the NHIS but in practice, membership is optional because there is no penalty for opting out of the Scheme. As at the end of 2014, active card-bearing membership of the NHIS was 38%, despite the fact that about 80% of Ghana’s population has ever registered with the Scheme. The gap between “ever registered” and “active membership” is seen as inefficiency that threatens Ghana’s march towards the attainment of universal health coverage and which raises concern among policy makers. Of particular concern is the case of Ashanti region where the NHIA is piloting capitation payment. At the onset of the pilot implementation, the NHIA indicated that the capitation payment was being piloted for one year, after which it would be rolled out in all regions. Three years into the implementation, the pilot continues in the region, a situation that has engendered various reactions from residents of the region, including providers, politicians and civil society groups (The Ghanaian Times newspaper dated 02/01/2012; The Daily Guide newspaper of 23/01/2012; The Daily Guide newspaper of 25/01/2012; Ghana News Agency: In the Daily Guide newspaper of 01/12/2012). Some of the issues they raised about the capitation payment policy include among others, falling NHIS-membership and enrolment renewal rates in the region.

### Previous studies on Ghana’s NHIS and capitation payment

A review of background literature on capitation experience revealed that studies on capitation payment and its effect on health care delivery are scanty in low and middle-income countries [12]. A study of provider payment method in a community-based health insurance scheme in Burkina Faso [13] revealed that capitation payment resulted in lower health worker motivation and negatively affected service quality and retention of enrollees. In Ghana there are studies on the association between health worker motivation and health care quality efforts [14] but these studies were not specific to the NHIS and its provider payment methods. Other studies on the Ghana NHIS have focused on perceptions and health seeking behaviour under the NHIS [15], reasons for people’s refusal to enroll with the NHIS [16], household perceptions and their implications on enrollment in the NHIS [17] and an evaluation of insured members perception and factors influencing their membership renewal decision [18]. A study of provider payments methods within the NHIS [19] did not address capitation payment because it was being implemented in only one region at the time of the study. The only studies on Ghana’s capitation payment method sighted in literature focused on knowledge, perception and expectations of insured clients under capitation payment [20] and the implementation challenges [21]. To our current knowledge, there is no study on the capitation implementation in Ghana that seeks to understand whether capitation payment influences insurance members’ renewal decision. The objective of this study was, therefore, to understand whether capitation payment influenced members’ decision to renew their membership with the NHIS. Findings from this study could provide guidance in shaping the policy debate as the NHIA plans to roll out capitation payment nationwide. It will equally provide guidance to other low/middle-income countries that are contemplating the adoption of capitation payment as a provider payment method in their health insurance scheme and also contribute to existing body of knowledge on factors that influence people’s decision to renew their membership with a health insurance scheme.

## Methods

### Study design

We applied a mixed method design for the study. We initially did a database review [Additional file 1] by analysing the NHIA central administrative data on NHIS membership for the period 2010 to 2014 from the Ashanti region where capitation payment is being implemented (intervention) and compared with those from Volta and Central (control) regions that have been selected by the NHIA as the next set of regions for capitation payment roll out. We also did a review of the literature to identify factors that could influence people’s decision to renew their insurance policy in order to inform the development of our interview guide. We then conducted individual in-depth interviews with residents in two districts of the Ashanti region, purposively selected for being the study districts of similar study, to understand whether capitation payment is a major factor in their membership renewal decision. Fifty (50) subjects were selected from the two NHIS district offices and the surrounding areas for the in-depth interviews. In each district, twenty-five interviews were conducted. Ten respondents were selected from each of the two district offices. We selected the first person seated on the front row as first respondents and subsequently, every fifth person, counting from the first, was selected until we got the ten respondents in each of the two district offices for the interview. In the surrounding areas, we selected every fifth house, moving towards the east from the district office; and residents aged 18 years and above were interviewed until we got the 15 respondents from the surrounding areas of each of the two scheme offices. Many of the respondents were NHIS card-bearing members. An interview guide [Additional file 2] addressing factors that could influence enrolment or membership renewal based on the literature review was administered. Respondents were asked for their opinion on the NHIS in general, including the capitation payment policy and factors that influence their enrolment and renewal decision. The decision to ask general questions, instead of capitation-specific questions was deliberately made to determine whether the capitation payment would come up strongly and spontaneously as a key factor influencing members’ decision to renew their membership.

### Study setting

Study of the administrative data on membership covered three regions of Ghana: Ashanti, Volta and Central regions. Capitation payment policy which is the subject of study was first introduced in the Ashanti region in 2012 and was therefore selected as the “intervention” region for the study. Per the NHIA’s implementation plan, the policy will be implemented in the Volta region in 2016 and in the Central region in 2017 and were, therefore, selected as control regions. The Ashanti region is centrally located in the middle belt of Ghana and has a total population of 4,780,380, accounting for 19.4% of the total population of Ghana of which 60.6% is urban (Ghana statistical Service, 2010). The region has 25 districts offices of the NHIA with 1,585,098 active NHIS-card-bearing members representing 34% of the regional population in 2014. The Volta region has a population size of 2,118,252, representing 8.6% of national population of which 33.7% is urban. It has 15 districts offices of the NHIA and insured membership of 581,305 representing 28% of the regional population as of 2014. The Central region has a population of 2,201,863 representing 8.9% of the national population. It has 13 districts offices of the NHIA and insured membership of 492,715 representing 23% of the regional population in 2014. The individual in-depth interviews covered two districts in the region namely, Subin sub-metro with a population of 174,004 and Ejisu-Juaben municipal with a population of 143,762. Subin is located in the central business area of Kumasi, the capital city of Ashanti while Ejisu-Juaben, with rural characteristics, is located at the outskirt of Kumasi. Both Subin and Ejisu-Juaben districts have NHIS office and membership coverage of 145,482 and 75,747, respectively, as of December, 2014.

### Data analysis

We did a trend analysis within and among the three regions to determine trends in new enrolment, renewal and overall growth rates over a 5-year period. Using SPSS (v. 20.0.0.1) we performed independent-samples *t*-test to compare the sample means of growth rates between Ashanti (intervention) and Volta and Central (control) regions. We subsequently performed the independent-samples *t*-test to compute and compare the sample means between Ashanti (intervention) and Volta and Central (control) regions. We did between subjects/group analysis to compare growth rates between Ashanti and Volta and Central regions as follows: (a) Ashanti versus Volta, (b) Ashanti versus Central and (c) Volta versus Central. Having determined differences between the sample means of the intervention and control regions to assess the significance level, we then calculated the effect size to determine how substantive our findings were. Data from the individual in-depth interviews were analysed using Atlas.ti. First, all the interview transcripts were read thoroughly and codes were assigned to quotes. The codes were based on factors identified in the literature search that could influence renewal decision. Subsequently, the codes were matched and generated into common themes that were used to categorize the factors as either being personal, scheme, provider, or capitation-related factors.

## Results

### Basic socio-demographic characteristics of respondents

A set of 50 individual subjects participated in the in-depth interviews (Table 1).

**Table 1:** Background characteristics of in-depth interview respondents (*n* = 50)

Characteristic	Frequency	percent (%)
Age (yrs)		
< 30	19	38.0
30–39	12	24.0
40–49	9	18.0
50–59	6	12.0
60–69	4	8.0
Mean age, y (SD)	37 (13)	
Sex		
Male	22	44.0
Female	28	56.0
Insurance status		
Insured	45	90.0
Uninsured	5	10.0

Source: In-depth interviews, 2014

### Trends in growth of membership enrollment and renewal

#### New enrollment

**Fig. 1:**
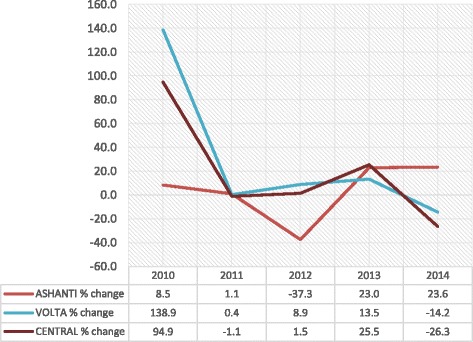
Trends in new enrollment growth rates: 2010–2014

The trend in new enrollment from 2009 to 2014 indicate that in the year 2010, all three regions recorded positive growth rates, with the Volta region recording 138.9% followed by the Central region with 94.9% and the Ashanti region with 8.5% (Fig. 1). In 2011, there was a drop in growth rate across all three regions. Ashanti dropped from 8.5% in 2010 to 1.1% in 2011. Volta also dropped steeply from 138.9% in 2010 to 0.4% in 2011 while Central dropped from 94.9% in 2010 to a negative growth of − 1.1% in 2011. In 2012, the year of capitation implementation in the Ashanti, the Ashanti region recorded a negative growth of − 37.3% while Volta and Central regions recorded positive growth rates of 8.9% and 1.5%, respectively. In 2013 all three regions recorded positive growth, with Ashanti moving from its negative growth of − 37.3% the previous year to a positive growth of 23%. Volta region moved slightly upwards from 8.9% to 13.5 and Central moved substantially from 1.5% the previous year to 25.5%. In 2014, Ashanti region maintained its positive growth with a slight increase from 23% in 2013 to 23.6% but Volta and Central regions went into the negative from 13.5% and 25.5% in 2013 to − 14.2% and − 26.3%, respectively. The trend as reported shows that growth in new enrollment across the three regions has not been stable over the years and among the regions. However, a *t*-test statistic result showed differences in mean growth rates between Ashanti (*M* = 30.15, *SE* 3.03) and Volta (*M* = 40.72, *SE* 3.10), the difference being − 10.56, CI [− 20.57, − 0.56] *t* (8) = − 2.435, *p* = 0.041; *r* = 0. 15; and Central (*M* = 47.38, *SE*6.49) the difference being − 17.22, CI [− 33.74, − 0.70], *t* (8) = − 2.404, *p* = 0.043; r = 0. 42.

#### Renewal

**Fig. 2:**
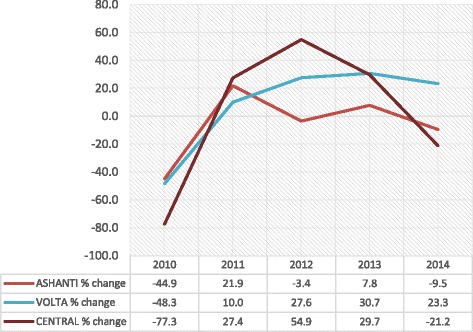
Trends in renewal growth rates: 2010–2014

All the three regions started on a negative growth rate in 2010 with Ashanti (− 44.9%) performing better than Volta (− 48.3% and Central (− 77.3%) regions (Fig. 2). In 2011, all three regions recorded positive growth rates. However in 2012, the year of capitation payment in Ashanti, the Ashanti region recorded a negative growth rate of − 3.4% while Volta (27.6%) and Central (54.9%) regions continued with their positive growth rates. In 2013, Ashanti recovered from its negative growth to a positive growth of 7.8%. Central region dropped from 54.9% in 2012 to 29.7% but the Volta region continued its positive growth and moved slightly from 27.6% in 2012 to 30.7%. In 2014, whereas Ashanti (− 9.5%) and Central (− 21.2%) regions experienced another negative growth rates, the Volta region remained in the positive although it dropped from 30.7% in 2013 to 23.3%. A *t*-test statistic result indicated that on the average, there were no significant differences in mean growth rates between Ashanti (*M* = 65.47, *SE* 6.67) and Volta (*M* = 69.29, *SE* 5.04), the difference being − 3.82, CI [− 23.1, 15.5] *t* (8) = − 0.456, *p* = 0.660; *r* = 0.03; and between Ashanti and Central (*M* = 50.51, *SE* 9.49) the difference being − 14.96, CI [− 11.8, 41.7], *t* (8) = 1.289, *p* = 0.233; *r* = 0.17.

#### Total active membership

**Fig. 3:**
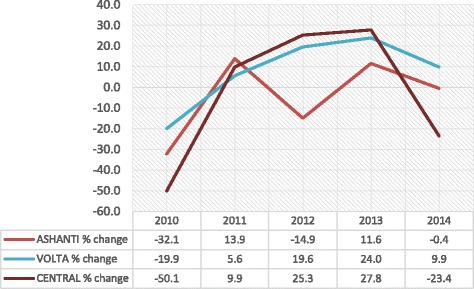
Trends in total active membership growth rates: 2010–2014

When we pooled the new members and the renewals together into total active card-bearing membership, we found that the pooled data show a similar trend as those of the new enrollment and renewal (Fig. 3). A *t*-test statistic result indicated that on the average, there were no significant differences in mean growth rates between Ashanti (*M* = − 13.76, *SE* 17.68) and Volta (*M* = 5.48, *SE* 5.50), the difference was − 19.25, CI [− 61.95, 23.45] *t* (8) = − 1.04, *p* = 0.329; *r* = 0.12; and between Ashanti and Central (*M* = − 6.47, *SE* 12.68) the difference being − 7.29, CI [− 57.47, 42.88], *t* (8) = − 0.335, *p* = 0.746; *r* = 0.14.

### People’s perceptions and membership retention

The individual in-depth interviews in the two districts revealed that personal, scheme and provider factors were the most important factors that influence one’s decision to enrol or renew their membership as noted in literature with capitation payment not coming up as a strong factor. We begin our presentation of findings with capitation factors which is the main focus of our study.

### Capitation payment factors

Our individual in-depth interviews with the 50 respondents in the two districts on factors that influence one’s decision to enrol of renew their membership indicate that capitation payment is not a key factor in membership retention decision-making. Respondents, however, expressed their opinions, both positive and negative, about the capitation payment policy and the implementation process.

On the positive side, some respondents mentioned that the choice of one preferred primary care provider (PPP) is a good innovation to improve quality of care in the health delivery system as may be inferred from the following statement: *“It is okay to choose one hospital because they will know your medical history. The quality of care is okay, there is no change.”* Other corroborative statements include the following*: “Capitation is okay because sometimes someone would go to the hospital and receive a drug, sometimes in the form of injection. If it is not working they would go to a second hospital and receive another drug or injection. Not noticing it is the same, they will take it and receive overdose, illiteracy is a big problem here,”; “Due to capitation the competition between hospitals has grown and therefore customer care at the hospital has improved”.* Commenting on provider shopping, a respondent had this to say*: “Capitation is good. People were moving from hospital to hospital, 3 times a week. Now with capitation, they go to the same hospital where they (providers) encourage them (patients) to finish the treatment*”.

On the negative side, some respondents who had a negative perception about the PPP concept noted that *“Capitation is very bad. That you can go to the only one facility you choose is bad. When you are dying and you have to go to the hospital quickly, it is very inconvenient”.* One other respondent who was not satisfied with capitation payment and the PPP concept stated that *“Capitation is not good, it should stop. You can only choose one hospital, and when you travel to Eastern or Western (regions in Ghana); you cannot go to the hospital over there. You have to travel back to your local hospital to get care.* Another respondent also lamented that “*Capitation is a problem. They (policy makers/NHIA) have to relook it. Sometimes you go to the hospital and your name is not in the file and then you don’t get help. Quality of care has also changed. At the facilities you don’t get all the medication you need and you have to go to the drugstore due to capitation.”* Others corroborated as follows: *“It (capitation) is becoming a problem. It is on trial, only in the Ashanti region and I would not recommend it for the whole country. When there was no capitation, there was no co-payment, now there is”; I am not satisfied with the quality of care, it is bad. I am not satisfied with the treatments. With capitation the quality of care should be good, but it isn’t. When the quality of care is bad, there is no need to renew membership”.*

Others with balanced opinion about capitation payment indicated that “*capitation is good. The way it is managed is the problem. The problem seems to be that providers are not well briefed on how it works. I don’t blame them. Education should be a lot. They (NHIA) need to get down to the very last person on the ground to inform, as well as service providers as the subscribers*”.

### Personal factors

As indicated earlier, personal, scheme and provider factors were key determinants of people’s decision to enrol or renew their membership with the NHIS. With regard to personal factors, the key dimensions were affordability of membership renewal fee, expressed need for health insurance, peer influence, subscriber expectations, and the concept of solidarity each of which could either positively or negatively influence one’s decision to enrol or renew their membership with the NHIS.

Regarding affordability, some respondents found the renewal fee of GHS14.00 ($3.50) per annum to be affordable for the reason that it offered insured persons free access to health care services for a whole year, as noted by one respondent in the following statement: *“indirectly you only pay around GHS1.00 a month and if you would not have insurance, fall ill and visit a hospital, you would pay more money than the amount you pay for insurance”*. They were therefore of the view that the renewal fee could not be a major factor to deter someone from renewing his/her membership. Other respondents also mentioned that health insurance protects them against the costs of health care as expressed in the statement: “*Insurance gives me peace of mind. When you are sick you don’t have to worry about financial constraints when you go to the hospital”.* However, few respondents did not feel in need of health insurance mentioned because (i) they do not fall sick often; (ii) they do not benefit by way of service utilization from the premium contribution they pay to the scheme, and (iii) that although they pay higher premium than others, they receive the same benefit as everyone else, as has been noted in other studies [22–26].

Peer influence was another dimension to the personal factors. It had a positive effect on membership renewal as some respondents mentioned that they were influenced by peers to enrol into the NHIS and renew their membership on regular basis. Other people who had insurance cover also influenced the uninsured to enrol and retain their membership with the Scheme. However, other respondents mentioned that they experienced negative influence on their enrolment and renewal decision from health care providers who had negative perception of the NHIS due to delays in their claims settlements.

People’s expectations also played a significant role in their renewal decision. Some respondents said their expectations were fulfilled while others experienced disappointment upon realization at the health facility that the NHIS did not cover every health condition: “*You would go to the hospital and they will tell you that these drugs are not covered by health insurance while I thought they were covered”.* Another respondent also had this to say: *“Two weeks ago, I had malaria, went to the hospital and had to pay for the blood test myself. At the moment you have to make a lot of co-payments, first it wasn’t like that, but now it is”.* With regard to the solidarity, some respondents were of the view that “*it is not fair to ask the rich to pay higher contribution*” while receiving the same benefit package rationalizing that rich people have the money to visit private hospitals where they would receive better services.

### Scheme factors

Under scheme factors, the benefit package was a major issue that influences people’s decision to enrol or renew their membership with the NHIS. Few people, however, said that the benefit package is extensive enough. They reasoned that expanding the benefit package could add to the sustainability challenges for the NHIS. However, many respondents perceived the benefit package as too limited in scope, as expressed in the following statement: “*It is not exhaustive enough; the number of drugs that is covered is limited. You would go to the hospital and they’ll tell you: these drugs are not covered by health insurance”*. Thus, respondents felt that there was no benefit of having health insurance when they have to pay for what they perceive to be better medication. Hence, they would rather pay for “good medication” than to pay insurance contribution. Many respondents were, however, positive about the attitude of the scheme staff, contrary to findings in other studies on the NHIS [25–28]. Respondents were also positive about the renewal process since: “*it is instant, clear, and orderly”*. However, consistent with findings from a study of factors influencing renewal decision [18], some respondents who were unhappy about the renewal process mentioned long queues and waiting hours and rampant system downtime as factors that affect the attractiveness of scheme to most people. As noted in earlier studies [25, 28, 29], as barriers to enrolment and renewals, some respondents were also not satisfied with provision of information on membership renewals lamenting that in order to receive good information; one has to go to the district office, which could be inconvenient for people who do not live close to the Scheme office.

### Provider factors

Quality of care was a key dimension of provider factors that influence renewal decision among respondents. Many respondents were satisfied with the quality of care they received from health care providers and as one respondent puts it *“the doctors and nurses take enough time for you and do what they can to help you get well quickly”*. They also mentioned the friendliness of the hospital staff towards them (insured clients) as some of the good qualities they found with the service provision. One respondent pointed out that “*if you have insurance the staff attend to you quickly”*. Some other respondents, however, mentioned that the quality of treatment depends on how promptly the NHIS settles health care providers’ claims. They mentioned that delays in settling providers’ claims affect the service they received, and as one puts it *“they will not treat you well, will not accept your card and will not even look at you”*. They further mentioned that the hospital staff pay more attention to clients who have money and are ready to pay-out-pocket than they pay to the insured. Others, however, said the service provision depends on whether the facility is a government or a private one. They observed that government facilities are overwhelmed with large number of patients and this affects the quality of service provided as evidenced in this statement by a respondent: *“in the private hospitals, there are less people so the doctors and nurses have more time for the insured. Sometimes they will ask for co-payments, but people are happy with that because of the quality of service”.*

## Discussion

### Database review

The trend analysis revealed that all the three regions experienced negative growth in both the new enrolment and renewal categories at one point or the other within the period 2010–2014, corroborating findings of a study in Senegal of a community-based health insurance scheme and membership retention [30]. But looking at the data from the overall effect, there is an indication that capitation payment had some effect in Ashanti region compared with the Volta region. However, since the effect is small and could not be sustained beyond 2012, it could be argued that other factors as have been noted in literature [15, 17, 24, 31, 32], might have contributed significantly to the drop in membership growth rates in the Ashanti region. This argument is supported by an earlier study that found that notwithstanding people’s negative attitude towards the capitation payment, a majority of survey respondents expressed their willingness to renew their membership card when it expires [33] and by our individual in-depth interviews which indicate that apart from the discomfort that respondents expressed with the capitation and its PPP arrangement, which is also consistent with findings by Pereira et at [34], capitation per se was not an important factor in their decision to enrol or renew their membership with the NHIS.

### Individual in-depth interviews

#### Capitation factors

Judging from the responses from respondents, one may say that a few respondents were positive about capitation. Their appreciation of the fact that capitation is a good innovation is good for the debate on the capitation payment policy. Their analytical thinking that capitation payment offers opportunity for continuity of care speaks to the fact that the communication messages about the positive attributes of capitation is making positive impact. It is however worth noting the reasons for seeing capitation as a bad policy by many of the respondents. The reason that subscribers who fall sick and attend facilities other than their PPP are refused treatment; co-payments demanded by some health care providers and the dispensing of perceived poor quality medication on the grounds of inadequate capitated rate and, or delayed reimbursement to providers are some of the issues that have the potential to erode subscribers’ confidence in the Scheme. The bad perceptions expressed by respondents are also noted by Owusu-Sekyere et al. [35], indicating how urgent the need is for the NHIA to address the issues to improve peoples’ confidence in the Scheme. This is importantly so because the issue of co-payment which, hitherto, has been treated as anecdotal, has been admitted by the Christian Health Association of Ghana (CHAG) in their 2015 Annual Report as real in the following statement: *“Providers are compelled to do balanced billing to make up for the difference between what the NHIA pays and what it costs them (the providers) to provide the care, thereby perpetrating out of pocket payments (co-payment) and pushing poor clients into catastrophic health expenditure”* (Christian Health Association of Ghana. Annual Report 2015, p57). It is also important for the NHIA to address itself to this concern because some respondents perceive the capitation payment policy as a good innovation and by addressing their concerns; the NHIA could move those who may be opposed to the capitation payment policy from their negative position to the positive position of those who are sympathetic to the policy. It is also noteworthy that most of the reasons underlying negative perceptions on capitation are borne out of inadequate information, education and communication to the public, especially the subscribers. The NHIA would therefore have to step up its information, education and communication programme to educate the public on the NHIS and the capitation payment policy.

#### Personal factors

Affordability as a dimension of personal factors inhibiting enrolment onto, or renewal of NHIS membership is consistent with findings in the literature which show that lack of money is the main reason for non-renewal of insurance [23–28, 36]. The literature has noted that in instances where membership is obtained while in employment, when people lost their jobs or retired, the barrier to renewal became bigger [22]. While this may be the case for non-retirees who may lose their jobs, it may not be so for retirees since they are covered under the exemption category of the NHIS. Inadequate information may therefore account for any such situation. It is also encouraging to note that people admit that they need health insurance, perhaps, more than health insurance needs them. This may be because, no matter how bad one may perceive the NHIS, one admits that it provides for them financial risk protection against the cost of health care services in times of need. These reasons, good as they may be from the perspectives of public health, could also become perverse incentives from the perspectives of the NHIS since most of those who may express the need for insurance and therefore enrol onto it, may also be those who will need health care services the more; and since every encounter with the health care system brings cost to the NHIS, their exclusive enrolment could add to the financial sustainability threats to the Scheme. While providers may be commended for the good testimonies from respondents who perceived them to provide good quality care, the reported negative influence from some healthcare providers coupled with the unauthorized co-payment in some health facilities also has the tendency to discourage people from enrolling and, or renewing their memberships as shown in other studies [17, 18, 24]. It must, however, be admitted that some respondents also had too high expectations of health insurance which led to disappointment and dissatisfaction with the NHIS. That notwithstanding, these unmet expectations of quality of service could contribute to non-renewal of insurance cover [18], especially for those who may not be in dire need of the NHIS.

#### Scheme factors

The issue about the adequacy or otherwise of the benefit package has remained a debate not only among subscribers but also among policy makers in Ghana. The dilemma has been how the quest for expansion of the benefit package could be aligned with the reported inadequate funding for the NHIS. Besides, the benefit package seem to have been loosely defined in the NHIS legislative instrument 2014, LI 1809 as “inclusion” and “exclusions”. This loose definition of the benefit package puts subscribers at the mercy of providers who define what is covered and what is not covered at the point of service delivery. Responses from respondents indicate that subscribers are not getting the full range of services they expect when they visit health care providers. These could also mean that subscribers do not have adequate knowledge of benefit package; hence, the reported co-payments at some facilities. As noted in other studies [18, 22, 24, 25, 27, 28], these are potential barriers to enrolment and renewals.

#### Provider factors

The key issues surrounding provider factors are the perceived poor quality of care and reported co-payment at some health facilities. But the issue of co-payment, since it is not formalized, could pose legal and ethical challenges as more and more people become enlightened on their rights and responsibilities under the NIHIS. It raises ethical issue if co-payment is taken for the reason that the NHIA does not pay health care providers on time as stated by a respondent. This is because, no matter how long it takes for providers to be paid, they still get paid but patients will not be called back to collect the payments they would have made. Tariffs are also negotiated and agreed upon between the NHIA and providers under the direction of the Ministry of Health. Agreed tariffs are duly approved by the Minister of Health and handed to the NHIA and its credentialed providers for compliance. It therefore becomes a challenge if subscribers, who are not party to the negotiations, are made to suffer the un-intended consequences of such negotiations. Co-payments are noted in literature [18, 26] to have negative influence on subscribers’ renewal decision and since the issue goes beyond the NHIA, the Ministry of Health may want to step up its stewardship role and put in place measures to address it.

Limitations of the study include the relatively short period that the administrative data was used for of the trend analysis (2010–2014); the convenience selection of the two districts and relatively small sample for the in-depth interviews which may be deemed not to adequately represent the views of residents in the entire region. Having acknowledged this limitation however, we are of the view that findings from the study provides a quick overview of important insights into motivations for enrolment and renewal decisions; and the diverse perceptions of the public could provide guidance to the NHIA to improve on the implementation process, having decided to implement the policy across the country.

## Conclusion and recommendation

We conclude that capitation payment had a small effect on membership growth rate in the Ashanti region compared with Volta and Central regions but that the effect was not sustainable since it was largely felt in year 2012 when the capitation payment was introduced. We also conclude that although many respondents have negative perception about the capitation payment policy, capitation payment is not a significant factor in their renewal decision and that, other factors may have played a much more significant role in peoples’ decision to enrol or renew their membership with the NHIS. Therefore, if membership retention is the only factor for deciding whether to scale up capitation across the country or to withdraw it, then results of the study present prospects for the scaling up of capitation payment in Ghana. The NHIA would, however, have to address itself to strategies that will enable members of the Scheme and also potential members to overcome the real barriers to enrolment and renewal of membership.

## Additional files

Additional file 1: NHIS membership dataset - new enrollment, renewal and total active membership data on NHIS subscribers in the Ashanti, Volta and Central regions of Ghana from 2009 to 2014. (XML 129 kb)

Additional file 2: Interview guide – interview questions posed to respondents during the individual in-depth interviews in the Ejuiso district and Subin sub-metro of the Ashanti region. (DOCX 18 kb)

Additional file 3: Ejuiso interview results – responses/statements from respondents in Ejuiso district of the Ashanti region. (DOCX 36 kb)

Additional file 4: Subin sub-metro interview results – responses/statements from respondents in Subin sub-metro of the Ashanti region. (DOCX 29 kb)

## Abbreviations

CHAG: Christian Health Association of Ghana
DRG: Diagnosis-Related Groupings
FFS: Fee-For-Service
NHIA: National Health Insurance Authority
NHIS: National Health Insurance Scheme
PPP: Preferred Primary Care Provider
