# Supplementary material for: Does a provider payment method affect membership retention in a health insurance scheme? a mixed method study of Ghana’s capitation payment for primary care
Source: BMC Health Serv Res. 2018 Jan 30;18:52. doi: 10.1186/s12913-018-2859-6 (PMC5789689; doi:10.1186/s12913-018-2859-6)
Supplement: Supplementary file 2 — Interview guide – interview questions posed to respondents during the individual in-depth interviews in the Ejuiso district and Subin sub-metro of the Ashanti region. (DOCX 18 kb) [file 12913_2018_2859_MOESM2_ESM.docx]

Interview guide

| MAIN QUESTION:  What made you decide to (not) renew health insurance? | | | |  |
| --- | --- | --- | --- | --- |
| **No.** | | **Purpose** | **Question** | |
| a | | Starter | Why did you become member in the past? | |
| b | |  | What were your expectations of health insurance?  Were your expectations answered? | |
|  | |  | **2.CHECKLIST** | |
| **No.** | V | **Bullet point** | **Question** | |
| 1 | □ | Understanding/ lack of education | How do you feel about the provided information on health insurance? | |
| 2 | □ | Benefit/need NHIS/alternative source of care | How do you feel about the need of having health insurance?  (why?) | |
| 3 | □ | Solidarity | How do you feel about the wealthy people paying for the poor?  - and the healthy people paying for the ill? | |
| 4 | □ | Peer pressure/ formal sector | How was your environment (family, friends) involved in your decision to be insured? | |
| 5 | □ | Reliability | How do you feel about the reliability of health insurance? | |
| 6 | □ | Quality of care | How did you experience the care as a member? | |
| 7 | □ | service provision | How do you experience the service as a member?  How do you feel about (the attitude of) care givers? | |
| 8 | □ | Benefit package | How do you feel about the benefit package? | |
| 9 | □ | Convenience NHIS | What do you think about the convenience of the district offices?  (location, opening hours?)  - How did you experience the process of registration? | |
| 10 | □ | Affordability | What do you think about the affordability of the registration fee?  What do you think about the affordability of the renewal fee? | |
| 11 | □ | Administrative challenges etc | How do you experience renewing your membership? | |
| 12 | □ | Information | What do you think about the provided information on renewal? | |
|  |  |  | **3.CAPITATION** | |
| **No.** | V | **Subject** | **Question** | |
| I | □ | Capitation | Have you heard about capitation?  (No -> Do you know that you have to pick one hospital?)  - What do you think of it?  - Why? | |
| II | □ | Provided information | How do you feel about the provided information on capitation? | |
| III | □ | Quality of care | Did you notice changes in quality of care after capitation?  - What kind of changes? | |
