# Supplementary material for: Does a provider payment method affect membership retention in a health insurance scheme? a mixed method study of Ghana’s capitation payment for primary care
Source: BMC Health Serv Res. 2018 Jan 30;18:52. doi: 10.1186/s12913-018-2859-6 (PMC5789689; doi:10.1186/s12913-018-2859-6)
Supplement: Supplementary file 3 — Ejuiso interview results – responses/statements from respondents in Ejuiso district of the Ashanti region. (DOCX 36 kb) [file 12913_2018_2859_MOESM3_ESM.docx]

**EJisu interviews**

**Interview No. 1**

a. Because of the costs of healthcare, it’s very costly. With insurance you have access every time, without financial difficulties.

b. First, it was free completely, now they often ask for copayments. The insurance people should act on that.

1. It is enough, most of the information he received via NHIS vehicle announcements or via the information centers.

2. The benefits are plenty. At times you fall sick and getting money is very difficult.

3. It is a good idea, the wealthy people should pay more to cover the poor, the rich should also be on board.

4. He decided for himself, not by peer pressure. Because in the future anything can happen.

5. Health insurance is always reliable, you can rely on it.

6. The treatment is okay, it is extensive enough.

7. The customer care, at times, is bad. The providers will ask for money, not official money, but for them personally to put in their pocket. If you don’t have money, they won’t treat you right.

8. It is okay how it is.

9. - It is okay, as well the opening hours as the location in terms of accessibility. He would appreciate it if they would be open in the weekends.

- When he came to register they gave him a due date on which his card would be ready, but when he came on the due date, his card was not in yet. They arranged him a temporary one.

10. It is expensive.

11. It is okay, smooth, he is okay with the processes.

I. He did not know about the term capitation but he did about the choosing of one hospital. It is okay to choose one hospital, because they know your medical history. The quality of care is okay, regarding capitation, there is no change.

**Interview No. 2**

a. When she was pregnant she decided to become member, now two years ago.

b. - She wanted to come to register, so they would provide her with access to healthcare.

- Yes

1. She understands the information, they will always tell you to become registered. She got information via the NHIS vans and the radio.

2. Without health insurance she would not have been able to bear the costs.

3. It’s okay, the wealthy people should pay for the poor.

4. A family member of her fell sick, then she saw the importance of health insurance and became registered.

5. In terms of reliability, it is okay. Even in the future she can rely on it.

6. It is okay, they have enough time for her.

7. They’ll ask you whether you are insured and if you’re not, they’ll advise you to do it.

8. She’s okay with the drugs she has been supplied with in the hospitals.

9. It is too far, she has to take a car. Opening hours 8-5 are okay. She would prefer if the office to be open on Saturdays.

10. - Pregnant so she did not have to pay, but considering the benefit you get from health insurance, the price is okay. When she got registered her husband had to pay though, which she thinks is a difficulty.

- It is okay.

11. The process is okay.

12. She knows when the card expires but that’s it, she did not know about the price until she is in the office.

I. She did not know about the term capitation but knew about the one hospital. She thinks it is good, because when she goes there she is treated well. She does not know much about the capitation.

**Interview No. 3**

a. In 2011, she came here to enable her to access health care, to get quality care, customer service.

b. They were met.

1. She received enough information on health insurance via the radio and on hospital premises.

2. Depends -> 6/7

3. The wealthy ones should pay more to help the poor.

4. Her parents advised her to do it.

5. Depends on how it is managed by the schemes, depends on how people understand the policy. For example, they may say it is not working, that will discourage people and reduce funds.

6/7. - Her child was sick and she needed drugs. So they went to the hospital and received drugs. She let the child use the drugs for three days and they did not work. So she went back to the hospital and received new drugs. After a while she realized they gave her the same drugs. So she went to the drugstore to buy drugs herself, this time another brand and this time they worked.

- Sometimes you have to make copayments. At times you’re being told to pay money. Two weeks it happened again, she was wondering whether it was legitimate.

- When you go to the hospital, people with money, without health insurance, will receive priority. This discourages because other people get priority.

8. It is not the best, but it is normal.

9. The office should be more visible, now it is like a hide-out place. It should be clear that it is a NHIS district office. Like with banks. If you see a bank you know it is a bank. The opening hours are good, the location is bad.

- The process is very slow. It should be like in the banks, you have the inscriptions: here is the atm, there you can do this, here you can do that.

10. - For her it is okay. Indirectly it is only 1 cedi a month.

- “ “

11. -

12. The information should be more. Many people go to the hospital and there they find out the card is expired and they should renew.

I. Capitation is okay, but sometimes when you travel, it is inconvenient. She traveled once to her mother’s place and then she got stomach problems. She went to the hospital there and they would not serve her, unless she paid. She had no money and it took her three days to get the money for treatment. Capitation is okay because sometimes someone would go to the hospital and receive a drug, sometimes in the form of an injection. If it is not working they would they would go to a second hospital and receive another drug/ injection. Not noticing it is the same, they’ll take, illiteracy is a big problem, and receive an overdose. When you have one particular doctor in one particular hospital, they know your history. The information was not good. I took my sick daughter to the hospital and there I heard I had to register for capitation. I didn’t really know how, they told me I had to go to the district office. But I had to go to school, so it was very challenging. Because my daughter was sick, I accepted it. The care did not change after capitation.

Additional comment:

When I wanted to register my daughter, I went to the office and they insisted that the husband would also come. But in Ghana it is not that the husband is obligated to stay with his wife when they have a child. My husband lives somewhere else. Some fathers fail to take responsibility. My husband wouldn’t come. I lied to them that he was traveling and that way I could get my kid insured. But some of the women leave without insuring their children.

**Interview No. 4**

a. It is helping the nation.

b. She wants them to be truthful. They’ll convince you to get insurance but later in the hospitals they won’t receive you. They tell you it doesn’t work, it is not active. When you register they’ll you it doesn’t work, it is non-active. When you register, they’ll give you a receipt, 3 months later you receive the membership card, meanwhile you paid for insurance, but you still have to pay for healthcare.

*Misunderstood the concept, receipt can’t be used to access healthcare.*

1. It was good, they came to the school tell about health insurance. I work at school.

2. Yes she really benefits from health insurance, recently she had an accident and health insurance really helped.

3. If you have the money you should pay more for the poor. They would probably not understand it or act on it but it would be good.

4. I spread that health insurance is good and they also advised her to get insurance.

5. Very reliable, she feels confident.

6. It was good, they take enough time for you.

7. I really like the way they handle patients. She praises them, they take very good care.

8. It is enough.

9. At first, they were not here but they moved. This location is better. The time is normal for offices. If they would open half days on Saturdays that would be good.

- It is going well, she has been here several times without problems, the waiting times are okay. They’ll give you a receipt so you can attend hospitals before your card is in.*

10. It is okay, if you wouldn’t have insurance and fall sick and go to the hospital, you would pay more money than the price you pay for insurance.

11. -

12. It is enough. They do public announcements.

I. I came to do capitation. I don’t know much, but I’ll will ask them to explain. (I asked if she knew that you have to choose one hospital, she knew so then I asked what she thought about that). I don’t like that you can only choose one hospital. When you travel, fall sick, if you choice is not there, you have to go back. I did not notice any change in quality of care.

**Interview No. 5**

a. In the beginning you would go to the hospital and pay before you received care. Insurance changed that. I became insured for quality care and to pay no money when you go to the hospital for care.

b. Insurance takes good care of me, every time I attend a hospital.

1. The information is very limited. Lots of people are not aware of what insurance consists of.

2. You can use health insurance to access healthcare at any point in time.

3. That’s the best, the wealthy should help the needy.

4. He heard about it on the radio, he did not wait for others to become insured.

5. Reliability is there, it has sustained for the 10 years he’s been insured and it has increased.

- It started with covering a few drugs, now it covers more.

- Now, the pregnant woman can go to the hospital for free.

6. In the hospital I go they take good care of me.

7. When he goes to the hospital, all it takes is showing your card and they will help you with everything you need.

8. When you would expand the benefit package with more sicknesses and drugs, the funds won’t be able to support it and insurance will collapse. I am okay with the benefit, but with time they should try to expand it .

9. Sometimes you have to wait very long, because there are problems with the process. The location is good. The opening hours are normal for offices. They should not be open in the weekends.

10. It is very cheap.

11. The process is smooth.

12. The information is not so much.

I. With capitation you have to choose one hospital so insurance can make advance payments. It is not good you can only choose one hospital because if you go you’re not supplied with the right drugs. It is good because your medical history is there. There has been changes in quality of care. Due to capitation the competition between hospitals has grown and therefore the customer care at the hospital level has improved.

**Interview No. 6**

a. It seemed wise to be part of health insurance, I just wanted to be part of it. When you fall sick, you don’t always have the money and with insurance you can go to the hospital.

b. - I expected good treatment.

- Yes, and they attend you quicker with health insurance with the health insurance than without health insurance.

1. I did not know much before I became insured. When I got registered I got the information.

2. It is okay. It really helped me and my kids.

3. It is good that the rich pay more because the poor sometimes can’t afford it, so it is good.

4. They all supported it. They all have it.

5. It is okay, you can rely on it.

6. It depends on the hospital, whether you go to a government or a private hospital. In the private hospital they take good care of you. Many people go to the government hospital, so often, they don’t have time for you. A lot of people don’t know that the private hospitals also accept health insurance. There is a perception that the private hospitals don’t accept it. At first they didn’t but now they do.

7. The doctors are fine but the nurses are too many. Some of the nurses are not qualified and don’t know how to treat people. Sometimes, less friendly.

8. It is okay, we don’t expect them to cover everything. Sometimes, the medicines are not covered and they’ll prescribe you the drugs to buy at the pharmacy. But you buy them for your own good. Sometimes, the medicines are expensive, but we can’t expect them to pay. We have to support them.

9. The location is good, but the compound is too small. The time is normal for offices. They should not be open in the weekends.

- The process is clear, with registration you have to wait 3 months but it is okay.

10. The price is okay, considering what you get for it, without you would pay a lot more.

11. -

12. The information was there, on the radio, the office people go there to explain, but also in the offices you get good information. They are very friendly and open.

I. I don’t like capitation. They told that when you travel you’ll be served at emergency at the hospital nearest to you. But only at emergency. When you need other care, you have to go back to your local hospital or change the capitation, you don’t go to the hospital as often as before. Information was enough, via the radio. The care did not change, it is just the same.

**Interview No. 7**

a. Insurance gives me peace of mind. When you are sick you don’t have to worry about financial constraints when you go to the hospital.

b. I expected that at any time I could use my card to access health care.

- Yes.

1. I’m okay with the information provision. I received information via the radio.

2. It helps you when you are sick. In times it happens that you fall sick while you don’t have money. With insurance they take care of you.

3. I’m okay with solidarity. The healthy ones should be on board to help the sick. And the rich should pay to cover the poor.

4. There was an occasion in my hometown. A man of 40-45 fell sick. I asked the man: “are you insured?”. “No”, said the man. Then the man was transported to the hospital and in every unit he had to pay. Because I did not want the same happening to me, I registered and also called my family to say they should become registered, because when you fall sick, your family won’t help you.

5. I believe in health insurance.

6. When I visited the hospital with my card treatment was okay for me.

7. I was told to pay for services not covered by insurance. But they’ll take care of the services within the package. The attitude of caregivers is fine.

8. I’m okay with it, because it is determined by law. The law knows why they choose to cover a certain package. When it is covered it is okay.

9. The process is about discipline. Over here, you have to cue. But I have never seen anyone crossing lines. The opening hours are normal for offices, even more, normal government offices close at 15.30h. Due to the tired making job, I don’t think people should work in the weekends. I like the premise, it is not nearby the car station, where it is very busy and people could be hit by cars.

10. The price is normal.

12. I am okay with the information renewal.

I. I accept capitation. I know they pay the hospitals in advance, know that the drugs are not part of capitation. When you go to the hospital a folder will be open in which your medical history will be established. I heard on the radio about it. I chose my preferred hospital in the hospital. There are no changes in quality of care, the way they take care of him is still the same, it is good.

**Interview No. 8**

a. Because of the benefits. Without health insurance you would pay a high amount of money.

b. - I expected excellent service.

- Sometimes, there are some problems with access. In some hospitals they won’t accept your card.

1. The information is not that elaborate. But because of the benefit you become member. I would like to see more information so we could tell our mothers who live in the villages about it.

2. I am not all that satisfied, sometimes in the hospitals they’ll tell you that the drugs are not covered and then you have to pay.

3. The rich should pay more.

4. Most of my family is insured.

5. It was reliable, but with capitation not always, when you are sick and go to the nearest hospital, they’ll tell you your capitation does not cover the service here and you have to go back to your own hospital. But you’re sick so you’ll pay.

6. It is good, just a normal process.

7. I go to a private hospital. And there the service is very fine. At the government hospital service is less. In the private you sometimes have to do a copayment, but I’m okay with it because of the service.

8. I’m not satisfied, it should cover more.

9. The building is too small but the location is good. The working hours are normal for offices. It would be good if they are open on Saturdays, maybe from 8 to 2. Today I couldn’t go to work because I had to go here.

- The process is tedious, you have to go from this table to that table. It should be simplified.

10. The price is okay, considering the benefit. I have diabetes and hypertension, so every month I go to the hospital for treatment and don’t have to pay.

12. The information on renewal is enough. You receive it in the office.

I. The information was not all that, because it was only in Ashanti, the information was small. The care did not change, is still the same.

**Interview No. 9**

a. So that the government would pay some of the hospital bills.

b. - At the time, I registered I was a minor, below 18, so I did not really have expectations, my parents made the decision. At 18 I stayed member, so I could access healthcare at the hospital.

1. It’s good, information was received via the television, information centers, via the community or radio.

2. It reduces all payment at the hospital. So I haven’t paid for anything, since she had health insurance.

3. It’s good, maybe you have insurance and you don’t fall sick, then your money can be used for the poor. Some poor don’t have insurance because they can’t afford it and then they fall sick and die. The wealthy people should pay.

4. Initially I thought I would not get, but then I realized everything can happen in the future.

5. I can rely on health insurance completely.

6. The treatments were good.

7. The attitude of care givers was good, they were friendly.

8. Yes, but last time I was treated, I needed a blood transfusion, which health insurance did not cover so she had to pay, but the rest of the treatments were all covered.

9. The opening hours and location are okay. It should not be open in the weekends. Then they should rest. The location is very accessible.

10. - It is not expensive, but they should not increase it.

- “ “

11. You have to cue, but not for too long. It is a clear process.

12. They tell you that when your card is expired for too long, the bills will increase.

I. Sometimes it is good, sometimes not good. Sometimes there is an emergency and you have to go to the nearest hospital. They won’t serve you unless you pay. Then You have to go back to the hospital you chose with capitation. It is good, because when you go to the hospital they have all the information on your history. The information was enough, within the community information was spread. The quality of care stayed the same.

**Interview No. 10**

a. I was registered by my mother. They registered to get access to healthcare.

b. People complain that they don’t get the medicines they need with insurance, they are restricted. Also, they are complaining that they let them wait and first pay attention to the people with money.

1. For me it is okay. Other people complain about the information. The people that work in the hospitals should be educated on how to handle with insurance.

2. It is good. My grandmother fell sick, went to the hospital and only had to pay a small amount of money, while without insurance it would be a huge amount.

3. It is bad, it should be mutual. It is not fair to ask the rich people to pay more money. It feels like cheating on them. People will pretend to be poor. They should decide on a moderate amount that everyone can pay. It is good that the healthy pay for the ill.

4. You have good and bad. For me health insurance is good, because of what happened to my grandmother. Capitation is bad though, because you can only go to the hospital you chose with capitation.

5. It is reliable, because when you have no money, you still have access to the hospital and receive good care.

6. In the hospital I work, they treat the patients with insurance well.

8. It is good, sometimes you don’t have money and you can still access healthcare. It is good, but if possible it should be expanded.

9. I was lost when I had to go here. I thought the office was in another place. I was searching for a sign but could not find it, so I had to ask someone in order to find the office. The opening hours are okay. In the weekends it would be good if they would be open for a few hours on Saturday. When the card expires in the weekend you can go then, you don’t know what might happen in the weekends.

- Some people say the process is difficult, you first have to cue and then go here, go there. Because of the long cueing some people decide to leave and their card expires.

10. It is good, if you consider the access to healthcare. Without health insurance you might pay more in the hospitals.

11. For me it was easy because she recognized where to go.

12. They should give more information at the media. They should go to the hospitals, communities and tell about the benefits and importance of renewal.

I. They should cancel it. You may only choose one hospital, before there were three. Say you leave Ejisu to go to the city and you fall sick, they have to get you back. When you go to the hospital with capitation, they’ll say you’re not in their system and you have to pay. At the emergency they will attend you but afterwards you have to pay. In the hospital I work, a lady came in who was seriously sick and she had to pay. The information we receive is not good, they should provide more information on capitation. They should, for a week to a month, go to hospitals, go to communities and intensively educate people on the benefits. It is only in Ashanti, they should implement it everywhere and let you choose one hospital in three regions.

**Interview No. 11**

a. To get access to free medical care.

b. - I expect them to take very good care in terms of health aspects, to provide all the needed drugs for all elements.

- The expectations partly came true, 50 percent.

1. They educate people, go from place to place, from community to community to give information.

2. It is so much encouraging, you can be operated for free without paying additional money. The scheme covers operations.

3. NHIS gives subsidy to the poor, they provide social security. It is good that the rich pay more than the poor because it provides access to the poor, the vulnerable.

4. All family members are covered. I came to collect their cards.

5. It is very reliable, the NHIS consists of hard working people, very energetic. They welcome everybody friendly.

6. Once you have the card and go to the hospital, they attend you very quickly. Insurance makes the work of providers easier.

7. They are not against it. They will always advise you to go and get registered if you haven’t done that yet.

8. Not all diseases are covered and then you have to go and buy the drugs outside the hospital. I shall be praying that one day it will cover all, then you’re free from the financial burden.

9. The building is in the right position. It is a central point in Ejisu. Everybody can access. The opening hours are okay. I would be very grateful if the office would be open in the weekends, Saturday and Sunday, maybe from 8 till 12.

- When the light are on the process is very fast, when the lights are off it takes longer to process.

10. I am a SSNIT contributor, what I pay is used to subsidize the poor & the pregnant. Sometimes they reduce the fee for promotion so more people are able to pay. But it is affordable.

11. -

12. They always come out to inform the people to renew every year. When you are in the hospitals they tell you your card is almost expired and you better go to renew it. The information is excellent.

I. It is not good. You may only choose one hospital. You registered for three hospitals. It would be better if you could use all three. Now, you have to go back to your local hospital every time. Some did not receive information. They go to the hospital and there they are told that their capitation is not there, consequently, they go from hospital to hospital to search where their capitation is, not knowing they have to register. A big problem is the illiteracy. Due to capitation you don’t get the right medication. The capitation is only in Ashanti, they should implement it everywhere.

**Interview no. 12**.

a. It was compulsory, a government policy. You had to do it. But it is good, when you fall sick without insurance and you don’t have money, you die. With insurance you can hospital without money.

b. - I expected you could go to the hospital without paying money. Initially, you pay a fee to become registered and then you don’t need money when you fall sick and go to the hospital and go to the hospital.

- Yes it is okay.

1. *Did not understand the question at first.* (Some people complain that it takes a lot of time to register because sometimes the lights go off and that delays the process. But they give you a temporary card.) The information is enough. They do announcements on the radio, in the press media such as papers, and on television.

2. The benefit is okay.

3. It is a government policy. But when you do have money and are able to go to private hospitals, it is up to you. But it is a good thing because it would help the authorities.

4. Everyone welcomed insurance.

5. It is reliable. There are some problems, but they are coming from the top, their ability to pay providers. But eventually the providers will get their money back. Below it is okay, the problems are at the top.

6. Some people complain that the drugs are not good. If the drugs are not covered you have to pay at the facility or they will give you a prescription and you have to go to a drugstore. They are not covered by insurance so I’m okay with it.

7. They are fine, they’ll attend you quickly. In some hospitals they are short of staff, but apart from that it is fine.

8. It should be improved. Some drugs are not covered, when you don’t have the money, you can’t get them. They should reconsider.

9. The location is good, it is within a big community. It is a government office, so the opening hours are normal. If Saturday could be added it would be good, to reduce the workload. Maybe from 8-12/1, because they also need to have time for family and other obligations.

10. It depends, when you’re not working it might be too much. Maybe the government should subsidize them so they are able to get registered.

11. It is fast, on the moment they’re working on it for me.

12. It is okay, they use notice boards to make announcements and advertize for it.

I. It’s becoming a problem. It is a trial, only in the Ashanti region. If we understand it, it will be okay eventually. People need to be well-informed, then they’ll accept it. Right now there is a lack of education. The capitation is okay, because I heard people to different hospitals at the same time, which makes the bills high. The quality of care is the same.

**Interview No. 13**

a. Because of the financial aspects. It is good to engage in health insurance, because you don’t know what could happen in the future. In case of emergency, you know you are supported.

b. - I expected quality health care/

- Yes it was, for me it was okay, but there are individual differences.

1. The information is normal, it is enough. You receive it at the hospital.

2. It is good.

3. It is good. We have less privilege. If the rich would pay more, it would help the economy. The health standard could be improved, as well as the economic status. The rich can help the poor.

4. They’ve been encouraging it. If you have money you can decide to join in.

5. (didn’t get the question) There are differences, but to me it’s no problem. The difference depends on the portfolio. Some people did not have much education on how to handle patients, policy holders. There is a need of public education. Some people don’t understand issues.

6. It is good. There is a good relationship because I’m a member. If you aren’t a member, the demands are high.

7. They are perfect, very caring.

8. It is good, it is enough.

9. It is good, the location is central, not far from anywhere. It would be good, if they were open in the weekends. I am supposed to be in class right now. Maybe on Saturday form 8-1 to benefit us.

- It is good, sometimes it is a bit slow, but the process is clear.

10. It is good, normal, everyone can afford it.

12. It is enough, but you have to consult the people in the office for information.

I. It is good, after 6 months you can change the hospital if you don’t like the service. They should change it, so you can choose two or three hospitals, than they would have to compete for your attention, to help the client, which would improve the service. Another thing is that they have your folder. When you go from hospital to hospital they may not know the medication you’re on or the right diagnosis. The information provided on capitation is not enough.

**Interview No. 14**

a. It is not all the time that you have money to go to the hospital.

b. - I expected quality healthcare.

- Sometimes the medication, they give you is not good, they’ll only give you paracetamol. Not all the medication is covered with insurance.

1. It is not enough, sometimes they’ll call you on the phone. Or you can get information in the office. The renewal should change to every two years. Sometimes you don’t have money and with one year being so short, you often realize too late that your card is expired.

2. It’s okay, we are registered under my husband. And they take good care of the ancient, they don’t have to pay.

3. They should pay more, the money for health care is not enough, not enough drugs are covered. When you don’t have money to buy the medication you need and which are not covered by health insurance, you’ll die.

4. *Did not get the question”. You can only register your direct family members. The daughter of my sister lives with us and I can’t register her as my daughter.

5. I feel comfortable about health insurance.

6. Very bad, the treatment is not good. If you pay cash, they will receive you first, if you have insurance they’ll let you wait. They tell you they don’t receive enough money for their workers. If you go with money they treat you proper. For drugs, most patients don’t even go with health insurance. They register so they can use their card as identity at the bank, not for health insurance.

8. It is not enough. They should include more diseases such as cancer and hepatitis B. The medication is really expensive, so a lot of people can’t pay for themselves and die.

9. The location is perfect. The opening hours are good, they are the normal office hours. It would be good if they are open in the weekends, because sometimes workers are not allowed to take some time off to go to the district office.

10. Both should come down, but they’re affordable.

11. It is good, they direct you at the door and then you have to cue. It is successful and clear.

12, It is better, you receive information at the office.

I. It is very bad. It is only in Ashanti. You can change it after 6 months, that’s too long. If I would be outside Kumasi, I’d be death and gone by the time I can change it. There is not enough information on capitation. The quality of care has changed. First, when you would go to hospital and leave, you wouldn’t be charged, now they charge you. You have to pay money, they’ll tell you, they don’t pay them and that’s why you should pay. Once a nurse was taking care of me and then she saw someone with money and I had to wait.

**Interview No. 15**

a. I am a SSNIT contributor, so it is mandatory to become a member. Also the money I pay to SSNIT is automatically conducted as a source.

b. - Quality healthcare

- Yes and no. Yes because when you’re member automatically get service. No because the service is not always quick. Sometimes you spend a whole day at the hospital. You wait and they won’t explain you why there is a delay. Just the other day, I went to the hospital to check my blood pressure and receive medicines. I showed my card, they told me to wait. So I waited one hour without being called. I went to ask the reason. They said they had to verify some information, but there was something wrong with the connection, it was slow. They should inform us on the reason why there is delay.

1. Once in awhile, they have advents on the radio or on the television. I heard there is a hotline but I never used it. I have two health insurance policies, I have a private one, because of the office I work and I have a public one, because I have two dependents who are not my biological children and are therefore not covered under the private insurance. In the office, you receive information, but it should be more proactive. Also, there should be a way, they could inform you your card is in. A year ago, I came in 5 months after I applied for cards. Two were in, the others not. After 3 months, I received the others. Meanwhile, every time I came in, I was disappointed. They should collect telephone numbers so they can send a message when your card is in.

2. It is quite beneficial. You can attend a hospital for basic care whether you have money or not. They’ll help you. When you don’t fall sick, your money will contribute to the care of people that do fall sick.

3. It is difficult to see whether people are rich. Can you see whether I am rich or poor? I don’t think that people who are rich would come to register for public healthcare. They have the money to go to private hospitals. When you go to the private hospital, they attend you quickly, they will offer you a cup of coffee while you wait, they have air-conditioning. The service is better. I don’t think they’ll come here to pay more for the same service. When they come here they pay the same for the same service. While, when you go to the hospital with money, you get speed service.

4. My wife and I took the decision. Because of the SSNIT contribution that is automatically conducted as a source of the NHIS and because of my dependents that are not covered under my office insurance. I did not receive advise from, for example, my father. I would rather give advice to the elderly.

5. Yes, on the ground that the coverage is nationwide. It used to be district-wide but now you can use it everywhere. The services between the hospitals differ, some services are poor or they won’t accept your insurance card.

6. Like I said, I can both attend the private and the public ones. The public hospitals are overwhelmed with the number of people which affects the type of service. In the private ones, there are less people and they have more time for you.

7.

8. It is not exhaustive enough, the number of drugs that is covered is limited. You would go to the hospital and they’ll tell you: these drugs are not covered by health insurance. I went yesterday and they told me that while I thought they were covered. They said I had to pay 15 cedi for the drugs.

The services in the hospital should be rated with for example a,b,c. When you come to register you should have the choice of service linked to what you are able and willing to pay. So, if you pay a higher premium you could get access to additional services.

9. I have to travel a few kilometers, so it depends on where you are from. It is not very far from the townships but if it is possible they should add a few locations. The working hours are normal office hours. It would be good if they add the weekends since some people don’t have time to come during the weeks. Sometimes it takes 2 to 3 hours in the office to receive what you came for, then you are away from the office for 2-3 hours.

- The process is orderly. Someone is outside to tell you where you have to go first. The challenge is that not all people speak Twi and that’s the language they start with in the office.

10. It is affordable, but it is relative to the person. A person who just turned 18 is no longer a dependent and when the person has no work he might have problems with affording the fee. But on average everyone is able to pay the fee.

12. Unless you come here, you don’t have information. They should submit more information, for example, on the costs, so you come prepared. More public education.

I. Capitation is restrictive. It restricts you where to go. You should be able to go to every hospital you want. It is good that they have your history but sometimes, when you are sick, you need to go to the nearest hospital and there they can’t attend you because it is not the hospital you choose with capitation. So you have to be transported to that hospital. Also, sometimes you’re sick and go to the hospital, but the treatment doesn’t work, then a month later you want to go to a second hospital for another opinion. With capitation they won’t attend you. The information on capitation is not good. You don’t get information unless you are in the office. When I came today they asked me on the spot to which hospital I wanted to go. So I had to decide on the spot. Then they gave me more information.

**Interview No. 16**

“Currently, for me health insurance is not so good. You pay your contribution, but the services are not good. Even drugs of 5 GHc are not covered. Last time, I was asked to buy a drug of 6 GHc myself. You ask yourself why am I member if I even have to buy the drugs of less than 10 cedi myself. You’d better take the money you have to pay to renew to pay for healthcare in the hospital.”

“Capitation, to me, is good. A lot of people were moving from hospital to hospital. They would go to one hospital, get medication. They won’t finish the drugs and go to a second hospital where they receive the same drugs, which they store at home. They would go 2-3 times a week for the same illness.”

“The benefit is not so much, you have to pay for drugs that costs less than 6 cedi. You’d prefer to keep the money you’d pay for renewal to use it for treatment.”

“Before the implementation, they gave you quite okay information.”

“The benefit is that some people can be operated for free. Though they maybe charge a little, considering you even have to pay for drugs that cost less than 6 cedi.”

**Interview No. 17**

“It is helping, without money you still have access to healthcare. Plus, the wives can access maternity care for free.”

“It is a major headache that insurance does not cover all drugs. If you have no money, you can’t pay for them. Some doctors will suggest to treat the patient outside insurance which means that insurance is not capable of providing the right treatment. A little time ago, my boy was sick and the doctor said: “If you rely on health insurance, the treatment wouldn’t help you.” For example, if you have malaria, the medication that is covered might not be strong enough for the type. Then they prescribe what is not within insurance.”

“Capitation is good. People were moving from here to there, from hospital to hospital, 3 times a week. With malaria treatment for instance you won’t see results until a few days. Normally, people would go to another hospital, now they go to the same hospital, where they encourage them to finish the treatment.”

“Ignorance is a problem, some people don’t know their card is expired, until they fall sick. Then they notice their card is expired and will rush to renew.”

“I don’t think that the amount of money you pay for the renewal fee is a problem. If you are able to pay the registration fee, you should also be able to pay the renewal fee, which is cheaper.”

“The information is not enough. Sometimes you see it in the print media but a lot of people don’t mind or are not able to read it. You have to go to the office for good information, but some people live 10 km away. Then it is not easy to stop by. Initially, they were always moving around with their vans, making announcements, but they stopped doing that. They should do that again, maybe once a month, raising conscious about renewal, tell people to check the dates.”

**Interview No. 18**

“In general, it is good, because in case of emergency you can access treatment when you don’t have money. There is a lot to improve though. There are services that insurance doesn’t cover. They should improve it cover all.”

“To me capitation is good. The way it is managed is the problem. The problem seems to be that providers are not well briefed on how it works. I don’t blame them. The amount of education is way too small. Also, providers receive 1.50 GHc a month per person which is not enough for the services, too small money. Education should be a lot. They need to get down to the very last person on the ground to inform, as well the service providers as the subscribers.”

“It can take a lot of time before insurance releases the money to the providers. The clients suffer. They’ll tell you, our money did not come in so you have to pay. It frustrates people, why would I renew my insurance if there’s no benefit.”

“People should be informed on when providers get money, so we can defend ourselves. Now we don’t know whether they are lying or not.”

**Interview No. 19**

“The policy is good. It helps the vulnerable, the children and the poor and provides equal opportunity for the community to access healthcare.”

“There are some things to improve. The scheme does not cover all the drugs, when you go to the hospital they’ll give you the general treatment, after which you sometimes don’t see improvement. Facilities should adjust the medication to the individual patient, no matter whether he is able to pay for it or not. Insurance should cover all drugs. People would be prepared to pay more for insurance.”

“Capitation is not important. The system should be open to everybody at any time. If I would happen to be hosted at another place, does that mean I have to go back?”

“Even, when you would have capitation in the Ejisu government hospital. There is an emergency and you would go to another hospital in Ejisu, which is the nearest at that moment, they’d ask you to go to your own hospital, in case of life and death. This happened to the wife of my younger brother. The facility they went to refused to attend them. There should be equal access to every hospital you want, as long as your membership card is valid.”

“People might decide not to renew, because of the treatment they receive. Often it is a routine, when you go to the hospital, they’ll give you paracetamol and vitamin B complex. If you have money you’d rather visit a private hospital where you can get various treatments. I prefer the private hospitals. Also, in public facilities, the way nurses and doctors receive patients with insurance ca be very bad.”

“Sometimes, they do promotions in which case they reduce the price of registration and renewal.”

**Interview No. 20**

“Former, it was better. Now it is not good, because of capitation. I don’t like that you can only choose one hospital. I’m not satisfied with the one I chose and want to change. The government does not bring in the money. The hospitals are complaining. Sometimes they don’t insure the expensive drugs, but the cheap ones instead.”

“The price they take might be too expensive for some people. Or forgetfulness could also play a part. (in non-renewal)”

“I had to bring my husband before I could renew. I was pregnant when I was first registered. Later I wanted to register my son under me but they did not allow it, because I had to bring my husband. He won’t come, certainly not when he knows, he has to pay money. The maternity care is very good by the way, it is all for free.”

**Interview No.21**

“Two weeks ago, I had malaria, went to the hospital and had to pay for the blood test myself. At the moment you have to do many copayments, first it wasn’t like that, but now it is. The lab tests are not covered, they used to be free.”

“Capitation is not good. If I would go to a different town and visit the hospital over there, I won’t get treatment. Even in Ejisu, you won’t be treated unless it is your own hospital.”

“I hear more and more people saying they won’t renew because they charge money, for tests. The day I went I was lucky I came with money.”

**Interview No.22**

“You have to do a lot of copayments. Initially, it wasn’t like that. I have been paying for the drugs.”

“Capitation is only in Ashanti and I would not advise it for the whole country. When there was no capitation, there was no copayment, now there is.”

“They decide not to renew because of capitation. When they want access to healthcare, they’ll say it is not there place. Also because of copayments, there is no reason to renew.”

**Interview No.23**

“Health insurance is good, at times you don’t have money at the moment you’re sick. Still you can go to the hospital. At times, certain drugs are not covered and you have to go and buy them yourself. At times, the renewal takes too long.”

“Capitation is not comfortable for me. I chose hospitals but they did not pick one of them for capitation and assigned me to another one.”

**Interview No. 24**

“I have a problem with health insurance. In the hospital they only give you paracetamol and multi-vitamins.”

“Going to one hospital is not good, I am not satisfied with the quality of care, it is bad. I want to change hospitals because of the quality of care. I am not satisfied with the treatments. Also, capitation is bad, because it limits accessibility of healthcare. With capitation the quality of care should be good, but it isn’t. In-patient treatment is okay, but the out-patient is bad with the introduction of capitation. When the quality of care is bad, there is no need to renew.”

**Interview No.25**

“Insurance is there to help. It is good. I’m okay with the coverage, but I don’t know much about the medication.”

“One hospital is good. It is bad when people go from hospital to hospital for treatment. That costs al lot of money. Choosing one place is good.”

“I don’t know about people that don’t that don’t want to renew.”

No.11: Market, female, 20-30, No English.

“Insurance helps us. I don’t know anything for improvement.” “Choosing one hospital is not good.” “I don’t know about people that don’t renew.”

**District manager Ejisu interview.**

1. Ways of providing information.

- Sometimes we organize gatherings/ durbars to provide information.

- We have people who represent the NHIS in the community, so called community agents, via them we can reach the community.

- We give messages to information centers, they’ll make announcements via speakers, like some kind of radio.

- The P.R.O. will go around with the van making announcements via the speakers.

- Every morning we educate people in the office.

- We will reach clients through providers.

- We will reach people through opinion leaders.

2. Why do you think some expectations are not met?

- Lack of education, some people don’t understand the packages.

- Some people don’t understand the concept and the issues.

- Some people think “Oh I’m sick, if the doctor gives me plenty drugs then he has seen me.” They think in quantity, they have the feeling that many medicines help them better than only one medicine.

- Sometimes providers ask for unjustified payments, the so called copayments.

- You have to wait three months before your card is in, some people think that when they come to register, they can instantly use the insurance to access healthcare. Some people think there is no waiting period but no receipt is accepted at the hospital, only the card or temporary card. The temporary card is given when the membership card hasn’t come in on time, otherwise not.

- Some people think 30 minutes is enough to go in and out of the district office.

3. Motivation to renewal?

When the card is valid they don’t have to pay at the hospital, when the card has expired, it is not valid and you have to pay.

4. Challenge to renewal?

- Financial difficulties. People could have no money to renew or pay the premium.

- People think it is a waste of time to come here and wait.

- Some people are so busy, that they can’t come here until they are sick.

- People forget the date of expiration.

5. **Why do people think capitation is bad?**

- Before capitation people would go to hospital number one and receive drugs. They would think the drugs they receive are too few and go to hospital number two.. etc etc. With capitation they don’t have that opportunity.

- People would complain “Oh but when I am far away….” But that is based on a misunderstanding because they can still go to a hospital when they are far away. Emergency is covered and emergence is defined in two ways: 1) When the person is unconscious and brought into a hospital, 2) When the person is too far away from its ppp, for example, because of a meeting. You do not even have to be unconscious, you can go there.

- Because of politics, the pilot is only in Ashanti. The ruling party has not many support in Ashanti, that’s why.

6. **Why is it a good thing?**

- It prevents provider shopping. The person is not going to use all the medication and still health insurance has to pay for it. Some people do take them all, the same drugs at the same time, which is not healthy.

- You have one hospital, your folder is there, your profile. They know where to continue. They can say: ‘Oh last time I gave you this medicine against malaria but it did not work, so this time I’ll give you another medicine.” If you go to various hospitals, you won’t have a profile.”

- The providers are paid in advance, when they get the money they can fund well.

7. Could it be possible to expand the package?

The scheme is only 10 years old, when you compare our system to other countries we are doing well. It is a social intervention, the money we get is too small to cover everything. If health insurance would cover everything, clients would have to pay a lot. Gradually, the package is expanding, slowly. People will complain based on the diagnosis they had. When the treatment is covered, they will be satisfied; when it is not covered, they’ll complain.

8. What could be a difference between Subin & Ejisu?

I can’t talk for Subin. In Ejisu there is constant communication between the district office and the providers. If we hear about copayments, we will go to the provider and tell them it is not acceptable. Maybe it is also because of the differences between urban & rural. In the rural area most people are farmers who walk distances, carry things. That is healthier, so the diagnoses might be minor compared to the diagnoses in the city and might be better covered. The cases could be different which is why people are less complaining.
