# Supplementary material for: Does a provider payment method affect membership retention in a health insurance scheme? a mixed method study of Ghana’s capitation payment for primary care
Source: BMC Health Serv Res. 2018 Jan 30;18:52. doi: 10.1186/s12913-018-2859-6 (PMC5789689; doi:10.1186/s12913-018-2859-6)
Supplement: Supplementary file 4 — Subin sub-metro interview results – responses/statements from respondents in Subin sub-metro of the Ashanti region. (DOCX 29 kb) [file 12913_2018_2859_MOESM4_ESM.docx]

**Subin Sub-metro interviews**

**Interview no. 1**

a. It cuts down the costs. At first you had to pay a high price. Now it’s subsidized.

b. - Health insurance was the order of the day. So he expected much attention at the health facilities.

- The expectations were met. The way they call you, you get better attention. Before, it was not good.

1. At the facilities you get information. They advise you to get insurance. Before he didn’t really get information. Especially, the information from the district offices is good.

2. Very good, before you had to pay too much. It was too expensive. Now it’s subsidized.

3. He did not really feel it was like that. He felt it is more mutual.

4. His mom advised him to get insurance, his little sister was sick and the costs were rising.

5. Good, you can attend health care very quickly. Even when you have no money with health insurance they help you.

6. Very good, no complaints.

7. They’re interested, do what they can do to help you to get you well quickly.

8. Good, he was feeling confident when he visited the clinic. It is extensive enough.

9. - It is okay, normal, good.

- He was very excited, did go for the temporary card.

10. Registration fee: - Normal not too expensive.

Renewal: - It should be constant, similar to registration.

11. Good, everything is in process when you get/wait here.

12. At the facilities you only get the advice to renew. For extensive information you should be at the district office.

I. “The translator did not even come to the end of his explanations, instantly he said it was not good at all” He wants to able to decide to which hospital he goes. ‘In case you get sick at night and the hospital is too far, what do you do then? The information he received on capitation was not sufficient, he was not satisfied. The quality of care at the hospitals did not change. Still good.

**Interview no. 2**

a. Hospital for free.

b. - He expected very good care at the hospital when you are member and quality service.

- At first his expectation were met, the service was good. But later, since the government changed, you had to start paying a lot for services health insurance did not cover. (Nothing to do with capitation).

1. The information provision was not good, you had to go to the district office to get good information.

2. Good, you can go to the hospital even if you do not have money. Sometimes you do not have money on you and you know you can still visit hospitals.

3. It is a good thing because it balances.

4. Everyone, family/friends was involved. They like insurance and advised him.

5. You can count on it very well. Even though sometimes you have to pay, you can rely on care.

6. At first it was good, now less good, because less is covered.

7. Very nice, when you are a member you do not get questioned. When you are not insured you get a lot of questions about money. ‘Even when you are dying they want to know whether you have money to buy a car, a house etc’. When you are insured they do not questions. They could inform you about what is covered and what not, but that’s it.

8. It is not extensive enough, often you have to pay.

9. It is okay, location, opening hours, all okay

10. Registration is cheap and the renewal fee also keeping mind that you have access to hospitals and care for a whole year.

11. Very quick, okay (renewal).

12. In the office you get good information, outside the office not.

I. “Again strong reaction, no need to introduce”.

Capitation is very bad. That you only can go to the facility you chose is bad. When you are dying and you have to go to the hospital quickly, it is very inconvenient. He did not get any information and you have to come to the office to get information/find out. Quality of care reduced because sometimes you need a certain doctor with a specialism. When you do not have the doctor in your facility and you go to another facility he will not help you.

“Again no need to introduce PPP, that’s the first thing they start about. That’s what they associate with capitation”.

**Interview no. 3**

a. When she went to the hospital for care it was too expensive. Health insurance drastically reduces the costs.

b. - She expected benefits, that she could easily attend hospital services, that it would improve the quality of care.

- She has not been a member for long, but till now everything is in place. However sometimes they charge extra money which is frustrated.

1. She’s aware of like 80% of the information on health insurance. She got the information when she went to the hospital (then uninsured). She saw card bearers, how they were treated and did not have to pay for anything, while she had to pay. She spoke to the people and they gave her information.

2. It helped so far, but she’s new and can’t really say much about it.

3. She feels it’s mutual.

4. When you are sick, family does nothing and that is an encouragement to get insured.

5. She feel it is reliable.

6. She is satisfied with the care.

7. They really take time for you. You have to cue when there are other members before you, but she is satisfied. The care she experience so far was good and the care givers were friendly and talked nicely.

8. It is okay, she feels happy about it. Does not know for sure yet, but for now she is satisfied.

9. - The number of district offices is good. Better have less offices and strengthen them. When you have more offices she thinks the quality is less. Also because of availability of employers. The opening hours were also good. From 8 till 5 and not at the weekends. Since employers also have family.

- Registration process was okay, not too hectic for her. She applied for a temporary card.

10 The registration fee is not expensive compared with what you get for it in terms of health care.

11. not applicable

12. She heard about renewal from others, knows little about it.

I. She heard that you had to report the hospital you preferred. She has not decided yet, because she wants to see which facility is the most convenient when she falls sick. She thinks it is a good thing because of processing claims.

**Interview No. 4**

a. She heard it was a good program. When you register you can go to the hospital with no money.

b. - She expected that when you fall sick, you don’t have to pay money.

- Expectations came true. She delivered in the hospital without paying any money.

1. At the facilities and hospitals you get good education on health insurance. They tell you why you should be insured. You also get information via radio.

2. It helped the family, saved money, protects against the costs of health care. There is need.

3. It is okay. The rich should pay more for health insurance.

4. They supported her and advised her to get insurance.

5. Reliability is there, but sometimes you go to the hospital and may not receive all the drugs you need and then you have to go to the drugstore.

6. It is good.

7. It is good, they talk nicely. The communication is good.

8. It is good. Sometimes it depends on the diagnosis whether it is covered. It should be expended.

9. - Location and opening hours are both okay.

- It was good, she applied for a temporary card.

10. - It is okay but if it could be reduced it is better.

- She thinks renewal should be free.

11. Renewal was good, orderly. No time was wasted.

12. You get information on the radio. They tell you to renew.

I. Capitation is a problem. They have to relook it, sometimes you go to the hospital and your name is not in the file and then you don’t get help. Information is not frequent. Sometimes on the radio or at the district offices. Quality of care has also changed. At the facilities you don’t get all the medication you need and you have to go to the drugstore due to capitation.

**Interview No. 5**

a. Because it would help my health.

b. - She expected it would help her and her family.

- yes.

1. It is good, okay. You get the information at the hospital and district offices.

2. It is good, She feels like she needs health insurance.

3. She thinks it has to be changed to equal.

4. The family/friends supported her, advised her to become insured. They also have insurance.

5. Yes it is good, she feels confident.

6. It is good, you get enough care.

7. They’re okay, friendly, she likes them.

8. It is okay enough, but to improve quality of care it should be expanded.

9. It is okay, when you come here, they’re quick and friendly.

10. - It should be free, but it is affordable.

- It should be free, but it is affordable.

11. It is okay.

12. Okay, you get information via the district office announcements, dates on the back.

I. It’s good. Money is too small for the hospitals. It’s okay that you only can choose one hospital. Information on capitation is okay. You get it from the radio. The quality of care did not change.

**Interview No. 6**

a. Because of the benefits, when you go to a hospital for care it is subsidized.

b. - He expected benefits, that it would cut costs, when you go to the hospital.

- Yes.

1. If you want good information you have to ask for it. Sometimes the information you receive from the hospital is different from the information you receive at the district office. For instance, about medication. At the office they will say it is okay and everything is covered while at the hospital you do not get all the medication you need and you have to go to the drugstore.

2. It is good, you need health insurance.

3. It is not like that, it is equal. The poor and the rich pay the same. The rich do not pay more. They pay 16 GHc and so do the poor. When I pay and I do not go to the hospital all year and someone else pays and goes to the hospital frequently, my money is used for them. But is okay, because I do not have to pay extra.

4. The advised him to register himself and get health insurance.

5. He has trust in the scheme.

6. For him the quality of care is really good. He has hypertension and when it is the date he has to go to the hospital the care is good and extensive enough.

7. It is good, when the date comes he has to go to the hospital they attend him quickly and are friendly.

8. It is okay, coverage is extensive enough.

9. - It is nearby in the centre. The opening hours are okay, only it should be open on Saturdays at least for a couple of hours, like 8-2 h.

- Registration is easy. Only on Mondays it is really busy.

10. - For him, the price is okay.

- They increased the price with two cedies but it is okay considering what you get for it.

11. It is okay, like 20 minutes till an hour and you are set.

12. There is not enough information. “He is well informed though, starts explaining renewal to me”. He tells the information on renewal when your card expires is really bad. After, there are no more renewal dates on your card and you have to apply for a new card. You hear about it when you come to the office, but they should submit the information, so you have the information before you go to the district office.

I. The benefit of capitation is that your medical report is in one place and therefore complete. There is a lack of information. He is well informed but other people are not. They don’t know they can only attend one hospital. They go to one hospital today and tomorrow to another. The people of the district offices should go to radio stations to educate people on capitation. The money the hospitals are receiving is too low and subsequently they sometimes do not give you all the medication you need.

**Interview No. 7**

a. You can go to the hospital when you do not have money. You receive quick service delivery. Drugs and medication are available.

- Expectations were met.

1. It was good he received information via radio stations, community announcements, local media, opinion leaders.

2. Yes he feels that he benefits from insurance.

3. It should be maintained.

4. Family members went and got registered. He saw the benefits, they informed him to register and so he went.

5. It is reliable, but there are some challenges, e.g. with the availability of drugs.

6. The care is good, extensive enough.

7. He is satisfied, but sometimes they are not friendly. The attitude is sometimes not good, they allow you to wait unnecessary. They give you the wrong directions in the hospital or sometimes the staff does not dare to come close to you, because they are scared of diseases.

8. It is good, though they should add more, because sometimes not all the things you need are covered.

9. It is accessible. He prefers that the office is also open on Saturdays, even if it is just for a few hours. He knows people that cannot come to the office because of work.

- The process is good. The set up is good with the direction signs.

10. - It is okay for him.

- It is okay for him.

11. It is okay.

12. It is okay. You have the back of the card as a reminder and you have announcements of the district offices.

I. Capitation has to be relooked at. It is not good. He wants to be able to go to more than one hospital. In case you’re traveling and get sick, you can’t go to the local hospital. The information is not enough, they should give more education on the radio and television. Capitation affected quality of care because sometimes they do not give enough drugs.

**Interview No. 8**

a. It was a compulsion, everyone should become member.

b. - Quick attention

- When the drugs are covered by insurance you get them for free, otherwise you have to pay. But there is quick attention.

1. To her it is good, if you understand it, it is good. Doctors and nurses give you enough information.

2. It is good, at first she did not have to pay for health insurance, now she does. Some drugs you pay.

3. If it qualifies. Some drugs are not covered whether you are rich or poor. If I’m not sick, I don’t go to the hospital and they take the money for someone else, it is fine.

4. She encourages her environment and they encourage her.

5. Trust, not that many, sometimes you pay for care.

6. It is enough.

7. They are very nice to her.

8. It is good, but not enough.

9. Location is okay. If they are running shifts the whole week, they should not be open in the weekends because in the weekends you rest. Otherwise, it would be okay if they’re open in the weekends, because some people can’t come during the week.

- It is good, if the network system work it is faster.

10. - Too high

- Too high, she’s got 5 children.

(At first she did not have to pay and then the government changed the rules and she had to pay (commends of an employer)).

11. It is good.

12. It is good, if you follow the instructions on the card it is good.

I. I have a problem with capitation. You can only go to one hospital. If there’s an emergency, you’re not close to your hospital. The information is enough. You receive it at the hospitals and district offices. The quality of care did not change.

**Interview No. 9**

a. When you fall sick you can go to the hospital without having any money. Health insurance saves money.

b. - It was beneficial

1. The information was good. You receive information via the radio, markets, community announcements and community agents that go from house to house.

2. She had to go to the main hospital for surgery and she did not pay anything. With no health insurance she should have paid.

3. It is okay, everybody should contribute, either big or small.

4. They supported her, the parents, the children, everyone.

5. It is reliable, when you have no money but you have the card, you can go to the hospital.

6. It is good, extensive enough.

7. It is good, they talk nicely.

8. It is good, it is enough.

9. It is accessible, they should not work on the weekends.

10. - It is okay

- It is okay

11. The process (registration/renewal) is okay. It is orderly. It is good that the work is divided, because one person could not do all the work.

12. It is good, because of the dates on the back of the cards.

I. In general capitation is good. But you should be able to choose more than one hospital. So in case you travel to the village, you could go to the hospital there. The information was not enough. Not elaborately enough explained. She got some information through people and agents but not enough. The agents of this office should go to their houses and explain.

**Interview No. 10**

a. The hospital is very expensive, when you have insurance it lowers the bills.

b. - I expected quality care.

- yes.

1. It was enough, you receive information at the facilities.

2. The benefit is perfect.

3. It is okay that the money of the people that do not go to the hospital is used for the people that go to the hospital often. Because you are helping each other.

4. They were okay with it, they were also insured.

5. Normal, she was not expecting much. But when you go to the hospital with the card you are treated well, while the people without insurance are not treated well.

6. I feel good, they took enough time.

7. Very good, normal, perfect. The service is good.

8. It is good and enough. She especially likes the free maternal care.

9. She lives nearby, so to her it is not a problem. The opening hours are fine, she does not think it should be open in the weekends.

- It was not difficult, you don’t have to wait long.

10. - It is moderate, everyone can pay it.

- “

11. It was not difficult, you don’t have to wait long.

12. The information was not good. You have to go to the office and ask for the information, then they’ll give you information.

I. At first, she heard from people about it, but she did not know much about capitation. Then she went to the office and got more information and thought capitation is okay. Though it is not good that you can only choose one hospital. She chose a local hospital, but when she goes to school in e.g. Accra and falls sick, she does not know what to do then. You should be able to choose more hospitals. The quality of care did not change.

**Interview No. 11**

a. It is a good program, a Ghanaian program. With insurance the care in the hospitals is cheaper.

b. He expected that it would save him bills

- it did

1. He got enough information via media, television and radio. And he looks for additional information on the net.

2. It is beneficial. When you don’t have insurance you need money when you go to the hospital. As long as you pay premium you are insured and you know you can go to the hospital.

3. It is a very good principle. It counts also for him and his relatives, they also benefit.

4. His environment embraces insurance.

5. It is reliable. All hospitals, public and even private embrace health insurance. So you can go to a lot of hospitals with insurance.

6. The quality of care depends on the hospital. In general, the private hospitals provide better care. Maybe because more people go to public hospitals it affects care.

7. Sometimes you have to wait a long time but the staff is trained and knows how to talk to you. So it is okay.

8. It is okay, it would be better if it is expanded, but it is manageable.

9. The number of offices should be extended to other areas, not only in the centre. The opening hours are normal for offices, so okay. It would be good when they are open in the weekends, because then some people could go in the weekend, which would reduce the cueing. But it is not bad that they are not open in the weekends.

- It should be improved. The waiting time till you receive your membership card should be reduced to 1-2 days.

10. - Very affordable

- “

11. The renewal process is okay, because it is an instant process.

12. They do not provide much information on renewal but you can read information on the card.

I. He does not like capitation for 100%, but it is acceptable. Only the number of hospitals you can attend should change from one to more. Because in case you are travelling and you fall sick. You do not have to go to the hospital you chose with capitation. The information on capitation was good for him. Via media. The quality of care did not change do to capitation.

**Interview No. 12**

a. It was more or less a compulsion. You did not really have an option.

b. - If you pay the contribution and you fall sick, you can go to the hospital and health insurance will pay your medical bills.

- Not quite, I expected that health insurance would cover every aspect, but it turned out that there were certain limitations. Certain sicknesses were not covered and you had to pay the bills yourself. So, the expectations are not really met.

1. It is too small. Like I said I expected that it would cover every aspect. They should tell more about what is covered. So you know what to expect and what not.

2. It depends, not all the drugs and sicknesses are covered. Also, when a woman has breast cancer she can go to the hospital and the treatment and medication will be paid. While, when a man has prostate cancer, it is not covered and he has to buy the medication himself. That is not good, if you pay the premium you should get the care.

3. (no need to ask the question) The money you pay goes into a pool. So when I don’t go to the hospital and someone else goes to the hospital frequently, the money goes to him/her. When I go to the hospital with something that is not covered, I don’t receive any money. When you contribute health insurance should cover it all.

4. He is working in an establishment. He had to be insured. The policy was that you should register and also register you wife and kids, the dependents. You had to provide the names. *Turns to Public Relations Officer for confirmation, he explains only the kids are the dependents, the wife should register herself*. See, the education is not good. I thought that my registration would automatically cover all, but my wife has to go to register herself.

5. It depends on how effective health insurance works. It depends on the money flow between health insurance and the providers. When the money flow is good, they will accept your card. But if the money flow is not good, if they did not get the money for serving you before, they will refuse taking your card at one point.

6. The nurses are doing their best but it also depends on the money flow.

7. *No need to ask the question* When they don’t receive money from health insurance it affects the hospital. They won’t treat you well. They won’t accept your card. They won’t even take a look at you. If you come in with a headache, they will ignore it.

8. Health insurance does not cover everything. Also, they try to cut down the costs. When you go to the hospital and you need medication they will provide you with a cheaper, maybe less effective medicine. The less effective drugs may not work. It depends on how much health insurance is prepared to invest. It would be good if they heighten the premium costs form let’s say 1 cedi to 5 Cedis, if then you receive more quality drugs and more treatments.

9. It is okay, it is in the centre and the opening hours are normal for offices. They should not have to be open in the weekends. - The processes of registration and renewal are good. It is just following the sequence.

10. He can’t really say something about it. He does not know who informs the government on what price is reasonable. But somehow the providers and health insurance should decide on it. If they can cover more and give better medication with more money, it is okay if the premium goes up. You want to go to the hospital and be happy, be better. If you don’t receive effective medication, you don’t get better. You should be able to go to the hospital for free, whether it’s your arm, leg or nose.

11.-

12. Renewal is clear because of the dates on the back, you know when you have to go to the office.

I. Capitation is okay. Your history is now in one hospital, when you go to the hospital it can be traced, while before you went from hospital to hospital. Information was adequate. Even before it was implemented you heard about it on the radio etc.; about that you have to choose one hospital. Quality of medicines had changed since capitation. The medication is set per diagnosis. But it may not be the best medication, but the less effective or medication that does not suit the person. It is not good because normally the doctor could choose the medicine that would work the best for the patient. Now they have to go with the medicine that is covered by health insurance and may not work for the person. The alternative is that they write a prescription and you buy it at the drugstore.

**Interview No. 13**

a. If you are sick it reduces some of the costs.

b. - Previous the costs were not so high when you needed health care. Now the costs are higher and you want to reduce them by having health insurance.

- Yes.

1. It is bad. You have to go to the market sites, hospitals to ask for information and then you receive it.

2. It is good.

3. It is good, they should maintain it. So that the ones that don’t have money can benefit from the people can benefit from the people that do have money.

4. She knows people who encourage other people, but herself, she is not encouraged.

5. Yes it helps, she is confident about health insurance.

6. The care is good.

7. It is good, they receive you very well.

8. It is good, enough is covered.

9. It is good, the location and time. If they could add Saturdays, that would be good, because workers can find time to go then.

10. It is both good.

11. It is good, nothing to complain about.

12. Also good. You know the actual time because it is on the back of the cards.

I. That one is bad. When she first heard about the capitation, she went straight to the hospital for information. They told her that she had to choose one hospital. Then she rushed to the district office to get capitation. It was very stressful since her baby was sick at that moment. She does not like it that you can only pick one hospital, because when you travel and you want to visit a hospital. You can’t. While when you used to travel, before capitation, you could use the health care in hospitals where ever you went. She did not get information on capitation. (Quality of care=) Some hospitals expanded which is good.

**Interview No. 14**

a. She went to the hospital without health insurance and the charged her 500 GHc. The doctor told her about health insurance and said she should get it. So she paid the 500 GHc and went to register herself.

b. - She expected the costs would come down and they did, she used health insurance several times.

1. It was okay, she received information via television, radio, announcements.

2/8. It is okay, but they should cover more.

3. It is good the rich should pay more.

4. They supported her. Her family and friends all have insurance.’

5. It is good, when you don’t have insurance there are financial difficulties. With insurance you benefit.

6. It is good, but sometimes the drugs are not covered and you have to buy them outside the facility in the drugstore. She expected that all drugs would be covered.

7. It is good, in the hospital she goes to they talk nicely and attend you well. She heart in some other hospitals it is different.

9. The opening hours & location are good. It should not be open in the weekends, then they should rest.

- It is good, but sometimes the clients are many and then you delay, for example on Mondays and Tuesdays.

10. It is okay, it has been reduced. The registration fee is more expensive than the renewal fee, so she had to pay less this time. But it is both affordable.

12. It is good because of the dates on the back of the card.

I. They should stop the capitation. She lived in Accra for a while and when she lived there she was able to go to every hospital she wanted. However, when she went came to Ashanti, she could only go to one hospital. In the past, she fell sick and went to the hospital, she received drugs, but was still sick, then she went to a second hospital, received drugs , but was still sick, then she went to a third hospital, received drugs and got better. That’s why you should be able to more than one hospital, for a second opinion. She did not receive information on capitation because she was not in Ashanti when it was implemented. When she came here, she heard about it.

**Interview No. 15.**

a. They told him about health insurance and how he would benefit from it. He became sick and had to go to the hospital for treatment. The total costs would be around 1000 GHc and thanks to health insurance he did not have to pay. He wants the benefit package to expand so more people would become member. He experience health insurance being a good thing and lowering the costs.

1. The information was okay. He received information via the radio, tv, internet, e.g. facebook.

2. It’s okay, it helped him and his family.

3. It’s should be maintained, the pro pore policy, the rich should pay more.

4. They supported him. His family and friends also have insurance.

5. Yes it helped almost everyone in Ghana. It should be maintained.

6. It’s okay, no problems, they get him everything he needs.

7. It’s okay, there’s customer satisfaction. The staff calms him, gets him a place to sit, they will even assist you.

9. It is good, the environment is good, easy access, easy transport. The opening hours are good, there’s no need to come in the weekends, the Saturdays and Sundays are to relax.

- It’s good, there’s a division of labor, everyone is working according to their schedule.

10. - It’s okay

- “ “

12. You have the dates on the back, you see when you have to renew.

I. Capitation is okay. It is good that you only have one doctor and one hospital. They have your history, they know the medication you’re on and the medication you need. The information is good but it should be increased. He got information again via the radio, television, Facebook. The quality of care did not change.

**Interview No. 16.**

a. You have to do it. It is important because of your health. Without health insurance you can’t attend the hospitals.

b. - The government should take care of the health of the people. They should take care of the needs of pregnant women, babies, elderly.

- Yes, but at times you get the drugs you need and at times you are asked to pay because they are not covered by health insurance.

1. The information is normal, it is enough but she would like to receive more.

2. It helped a lot. It helped her family and friends. She really appreciates insurance. The government should continue to help the patients, supply drugs.

3. It is good, not all people can afford the drugs. It is important that the government implemented health insurance to help people that can’t afford it.

4. They accept it, everybody is insured.

5. It is good, it has helped a lot.

6. Private and regular, they both accept the insurance card and give you a good diagnosis.

7. They are all good to you. As long as you are holding a card, they will attend to you. When you don’t have a card, they advise you to get insured because otherwise you have to pay.

8. You need more. Not all diseases are covered. Often the card is not capable to pay the whole treatment. They will tell you, it covers this and that but not that and then you have to a percentage yourself.

9. It is good, location & time. They should not be open in the weekends, maybe for the workers, but in the weekends you have to rest, otherwise it is not good.

- It is good, orderly, at times you have to wait too long, the cueing is too much. They should do something about that.

10. - it’s okay

- “ “

12. It’s on the back of the card. You know the time you have to go and renew.

I. I don’t like the capitation. It is bad, you can only go to one hospital. When you travel or visit someone or relocate you can’t go to the hospital there. The information is bad, it is not enough. The quality of care did not change after capitation.

**Interview No. 17**

“Health insurance is not always reliable, sometimes they do not accept your health insurance. You have to pay for some of the care or medication.”

In reaction to renewal:

“If I feel okay, I don’t check my card. If I’m feeling energetic and I’m working, I’m not going to renew. When you feel sick or weak and you want to go to the hospital, then you may realize your card is expired and you have to renew.”

“The opening hours are not very convenient, if you want to go and renew your card after work, it’s 5 o’clock and the office is closed. If they would be open in the weekends, maybe just for a couple of hours, than workers can go there.”

**Interview No. 18**

“I don’t have health insurance, because I’m not the type that falls sick a lot. Also, we have a private doctor who takes care of the family. My mom is sick though and she has the health insurance because otherwise the costs would be too high.”

“When health insurance was first starting it was very good, very beneficial. You would go to the hospital and get good care, the right medicines. It covered a lot more. Back then the government took good care of the people. Nowadays I see no benefit. When you go to the hospital with stomach ache, they’ll give you Paracetamol, when you have something with your head, they’ll give you Paracetamol. If you want good medication they’ll ask you to pay for it. A while ago my mom was suffering from pressure, so we went to the hospital and got medication. My mom took the medication and a day later she started complaining, so I looked at the box of the medication and it turned out to be expired for two years.”

“At the moment, the cash and carry is more beneficial, because you get good medication, good care. Right now, I see no benefit in health insurance. First it was better.”

“The capitation is not good, it should stop. You can only choose one hospital, but when you’re travelling to eastern or western, you cannot go to the hospital over there. You have to travel back to your local hospital to get care.”

**Interview No 19**

“Sometimes, health insurance is good, sometimes, health insurance is bad. Sometimes you don’t get the good drugs. If you have money to buy for health care the service is much better. You get good care, good medication. For me there is no need to have health insurance. If you have money, you get good services.”

“Health insurance is a good thing because it can reduce huge costs.”

“Capitation is not such a bad idea. When you only attend one hospital, they know your history and can give you good services.”

**Interview No. 20**

“When you have health insurance and you go to the hospital. They won’t give you the good medicines, you have to pay to get the good medicines. When you don’t have money they will prescribe you medication which you have to get it at the pharmacy. When you don’t have money you get not good medication.”

“First, they won’t take money and just give you the good medication. Since two years, 2012, they started to collect money.”

“You have to have capitation before you can go to the hospital. That’s not good. When someone form Accra comes to Ashanti and he falls sick, he first has to register for capitation here before he can attend a hospital here.”

“Every year you have to renew. The dates are on the back of the card. Maybe some people are not in time to renew because they don’t have money on the date they have to renew.”

**Interview No. 21**

“Insurance is very good, it helped so many people. On the moment I am not insured because I lost my card, but she will get a new one within this week.”

“The kind of drugs given to the people with insurance are not good.”

“Capitation is not good. At times you may not be in town, when you’re traveling and then you can’t attend a hospital.”

Renewal

“The drugs you receive with health insurance are not good. They only give you paracetamol and you have to buy the rest yourself. So then there is no need in having insurance and no need for them to renew.”

**Interview No. 22**

“The way they do is not proper. You go to the hospital with insurance and they will tell you the medicines are not covered and you have to buy them yourself. Before it was better.”

“For me there’s no need in having health insurance.”

Non-renewal:

“This government says you can only choose one hospital, people don’t feel good about it. It is a restriction. They’re not allowed to go to more than one hospital.

**Interview2 No. 23**

“It’s good, I don’t have enough money to pay the hospital bills. With insurance, it’s no problem.”

“If you go with money they do serve you well, with health insurance they don’t serve you well. When you have money you get all medicines. With health insurance you get only 1 a 2 medicines. You don’t get all the medicines, and you don’t get all treatment.”

“People do not renew because of the treatment you receive when you’re insured.”

“Sometimes the attitude of providers is not good towards people with insurance, but often it is good.”

“Capitation is not good, it does not help. You have to pick a hospital in this town. When you travel to, for example, your hometown and you fall sick and you don’t have money for transport back home you die.”

**Interview No. 24**

“At first health insurance was very good, very beneficial. But since the new government in 2009, she hasn’t seen any benefit from health insurance. In the hospital they don’t give you the right medication, only paracetamol, ibuprofen. They will prescribe you the good medicines and you have to go to a pharmacy to buy them yourself.”

“Capitation is not good, you have to choose one hospital. If you’re not in the environment you have to go back to where your hospital is, that’s not good for your health.”

Non-renewal

“People realize that they don’t get good medicines. People don’t like capitation. The new government showed the bad aspect of health insurance.”

“Also on the radio she heard hospitals were withdrawing from insurance, because they don’t receive their money. The money which is collected through 2.5% VAT/SSNIT should go directly to the NHIA and must only be used to pay the providers. Officials take some of the money and not all goes to the providers.”

**Interview no. 25**

“It is good for the country, it helps poor people to access health care.”

“The quality of medicines is not good. I don’t bother about getting insurance. The medicines are low-budget like paracetamol.”

“Capitation is not good. If the hospitals don’t have enough money, they won’t work. The hospital in this neighborhood is too small to have responsibility for all the people.”
